# Development of a portable toolkit to diagnose coral thermal stress

**DOI:** 10.1038/s41598-022-18653-3

**Published:** 2022-08-24

**Authors:** Zhuolun Meng, Amanda Williams, Pinky Liau, Timothy G. Stephens, Crawford Drury, Eric N. Chiles, Xiaoyang Su, Mehdi Javanmard, Debashish Bhattacharya

**Affiliations:** 1 Department of Electrical and Computer Engineering, Rutgers University, Piscataway, NJ 08854 USA; 2 Microbial Biology Graduate Program, Rutgers University, New Brunswick, NJ 08901 USA; 3 Department of Biochemistry and Microbiology, Rutgers University, New Brunswick, NJ 08901 USA; 4 Hawai’i Institute of Marine Biology, University of Hawai’i at Mānoa, Kaneohe, HI 96744 USA; 5 Metabolomics Shared Resource, Rutgers Cancer Institute of New Jersey, Rutgers University, New Brunswick, NJ 08901 USA; 6 Department of Medicine, Division of Endocrinology, Robert Wood Johnson Medical School, Rutgers University, New Brunswick, USA

**Keywords:** Ecophysiology, Predictive markers, Electrical and electronic engineering

## Abstract

Coral bleaching, precipitated by the expulsion of the algal symbionts that provide colonies with fixed carbon is a global threat to reef survival. To protect corals from anthropogenic stress, portable tools are needed to detect and diagnose stress syndromes and assess population health prior to extensive bleaching. Here, medical grade Urinalysis strips, used to detect an array of disease markers in humans, were tested on the lab stressed Hawaiian coral species, *Montipora capitata* (stress resistant) and *Pocillopora acuta* (stress sensitive), as well as samples from nature that also included *Porites compressa*. Of the 10 diagnostic reagent tests on these strips, two appear most applicable to corals: ketone and leukocytes. The test strip results from *M. capitata* were explored using existing transcriptomic data from the same samples and provided evidence of the stress syndromes detected by the strips. We designed a 3D printed smartphone holder and image processing software for field analysis of test strips (TestStripDX) and devised a simple strategy to generate color scores for corals (reflecting extent of bleaching) using a smartphone camera (CoralDX). Our approaches provide field deployable methods, that can be improved in the future (e.g., coral-specific stress test strips) to assess reef health using inexpensive tools and freely available software.

## Introduction

Having knowledge of coral health before the demise of reefs through bleaching can allow interventions to be enacted (e.g., restricting fishing, halting local development, or limiting tourist activities) to provide wild populations the time to potentially acclimate to environmental stressors^1^. Coral conservation using molecular data relies on basic knowledge about how the holobiont (i.e., the coral animal and its microbiome comprised of dinoflagellate algal symbionts, prokaryotes, and viruses) functions to allow identification of targets for analysis, or manipulation. However, the transition from a lab-based understanding of coral stress markers to field applications aimed at diagnosing and monitoring coral health has proven very challenging, with many approaches focusing on the photosynthetic activity^2^, cell counts, or chlorophyll *a* content^3^ of algal symbionts. These efforts include the use of small treatment tanks to control light, temperature, and waterflow conditions, followed by measurement of the photosynthetic efficiency of photosystem II using a pulse amplitude modulated (PAM) fluorometer^4–6^. This powerful method may be difficult to apply under field conditions, although approaches such as DIVING-PAM have proven useful in field deployments. A potentially more substantive issue is the time needed to do the coral stress treatments prior to gathering the PAM data. Regardless of the approach taken, the standing genetic variation of coral animals, combined with their complex biotic interactions with the microbiome under fluctuating environmental conditions will produce a panoply of holobiont responses that may prove difficult to summarize using one or a few stress markers^7–10^.

Healthy corals are expected to exhibit a broad array of natural biological responses under normal conditions, and only when they are stressed for prolonged periods due to local heating events or other insults do we expect stress markers to rise above the background "noise" inherent in complex systems^11,12^. Achieving the level of sensitivity required to assess coral stress prior to bleaching is the gold standard for field applications and recent data suggest that gene expression, proteomic, and metabolomic markers are promising in this regard^12–15^. These measures of stress however require specialized equipment and may prove costly or challenging to apply at remote reef sites that lack lab facilities or other needed infrastructure (these issues can, of course, be addressed over time). Therefore, given the current state of coral research and conservation needs, we explored the use of simple tools to assess coral biology and health that would be applicable worldwide and provide a more general assessment of coral stress “syndromes” rather than relying on specific gene or protein markers. We combined controlled lab experiments with field sampling to test the utility of diagnosing coral health using a well-characterized, widely available, and inexpensive test designed for humans that targets metabolites and proteins present in conserved animal pathways. We also designed a 3D printed smartphone holder and image processing workflow to enable users to easily and accurately read and process the results from the test strips (TestStripDX).

As an additional resource for field monitoring, we developed a system to simplify the collection of coral color scores, which is a minimally invasive and widely used method to approximate the extent of bleaching (i.e., loss of algal symbionts)^11,16^. Computer vision and image processing were utilized to rapidly and accurately determine color scores with an easy-to-use platform (CoralDX). Computer vision is an interdisciplinary area of research that generates a high-level understanding from digital images or video and allows collection of massive amounts of image data^17^. Traditionally, scientific tools such as ImageJ (https://imagej.nih.gov/ij/) have been applied for image creation and processing. However, many of these tools are manually operated and selection of specific areas of a sample for analysis has proven difficult and time consuming. Given these issues, our method was designed to accelerate and simplify the measurement process. Specifically, detectors were trained to automatically measure color scores based on the YOLOv4 computer vision model^18^. After detection and segmentation, image processing was used for the latter to isolate coral nubbins in the image field to measure the red, green, and blue (RGB) values. Measurement results from our computer vision software and the traditional ImageJ approach, were compared and we verified that the automated YOLOv4 method provides equal or superior results and can be used without the need to purchase commercial software.

## Results and discussion

**Figure 1:**
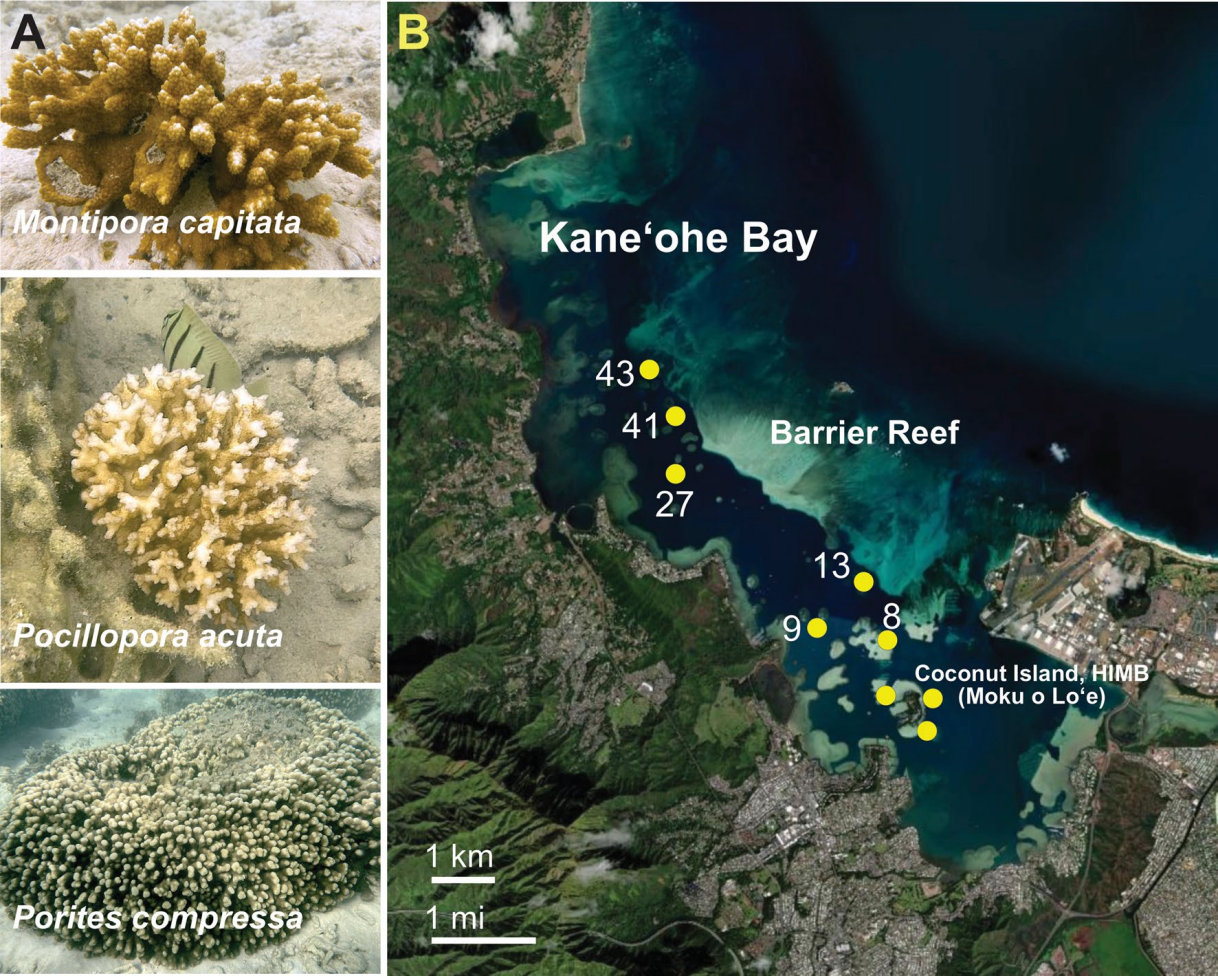
Analysis of Hawaiian corals. (**A**) The three targeted species in Kāneʻohe Bay. Images created by D. Bhattacharya. (**B**) Sites of wild coral collection (marked by the yellow circles) in Kāneʻohe Bay in June 2021. Nubbins from two colonies (n = 3) of *M. capitata* were collected from six reefs: Reef 8, 9, Reef 13, Reef 27, Reef 41, and Reef 43 and from three sites near the Hawaiʻi Institute of Marine Biology (HIMB) on Coconut Island (Moku o Loʻe). This image was adapted from the Pacific Islands ocean observing system (https://www.pacioos.hawaii.edu/projects/coral/)^33^.

To assess coral holobiont health, we used the commercially available Urinalysis strips (Accutest URS-14 100 strips Urinalysis Reagent Test Paper; ca. $11–35 USD/ bottle) that are designed to detect disease markers in humans, potentially indicating diabetes, metabolic abnormalities, liver diseases, kidney function, and urinary tract infections. There are ten tests on each strip that provide an initial assessment of health using the following standardized markers: leukocytes, nitrite, urobilinogen, protein, pH, blood, specific gravity, ketone, bilirubin, and glucose. To apply this test to coral extracts, we used ambient and stressed nubbins in a comparative approach to identify trends in the data. We presumed that some of the tests are likely not applicable to corals, whereas others such as ketones and leukocytes, which target conserved animal pathways (see below) could prove useful. Samples from three different coral experiments (two bleaching experiments and one environmental survey) were used to assess the response and utility of the different tests on each strip. The first bleaching experiments was done in 2019 on two Hawaiian species: *Montipora capitata* (stress resistant) and *Pocillopora acuta* (stress sensitive); this research has been previously described in detail^11,12^. Briefly, three *M. capitata* colonies (different genotypes) were selected, with samples collected from each in triplicate at three timepoints (referred to as T1, T3, and T5) that span a period of prolonged thermal stress (16 days). The second bleaching experiment was done in 2021 and included nubbins from *M. capitata*, *P. acuta*, and *P. compressa*. These corals were maintained for 9 days under heat treatment or ambient conditions, with samples collected at the beginning and end of the experimental period from both conditions (see Methods). Finally, the third experiment was done in June 2021 on wild colonies of *M. capitata*, *P. acuta*, and *P. compressa* (Fig. 1A) from Kāneʻohe Bay, Oʻahu, Hawaiʻi (Fig. 1B) and analyzed to assess the extent of natural variability in the test strip results. It should be noted that the test strip results from each experiment are independent (i.e., analyzed separately to measure relative differences within the test population) due to differences in tissue freezing time, storage length, and handling, that affect the results.

### Portable device for test strip analysis

**Figure 2:**
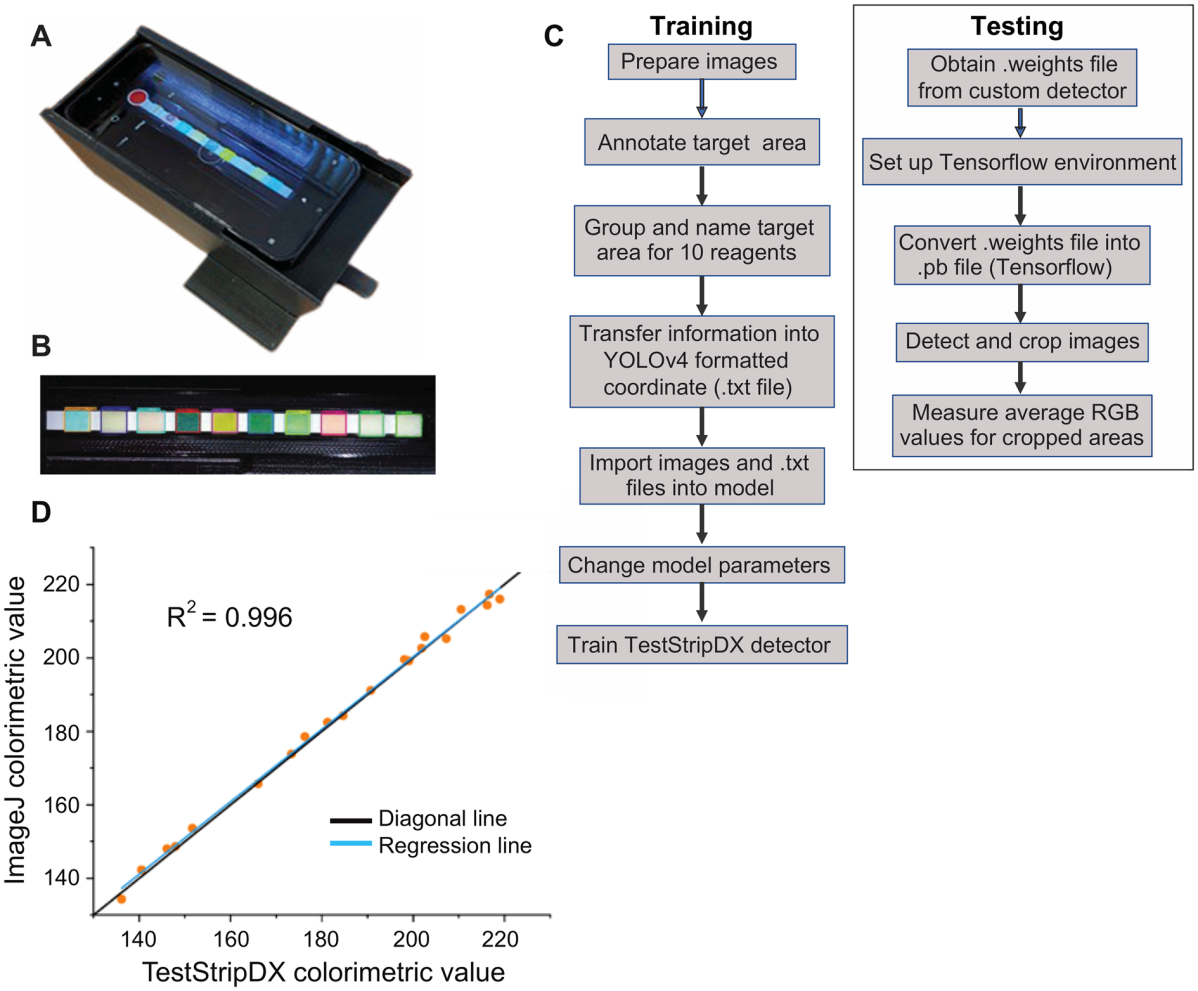
Portable instrument for test strip analysis. (**A**) The 3D-printed phone holder used to analyze the test strips. (**B**) Example test strip. (**C**) Flow chart for the training and application of the machine learning method (TestStripDX) used to analyze test strips. (**D**) Comparison of test strip colorimetric measurement values produced by TestStripDX and ImageJ, for a set of representative images (Table S1). The enforced diagonal matches very closely to the regression line.

To facilitate field testing and allow accurate measurement of test strip results in the R, G, and B channels, a combination of 3D printing technology and computer vision was applied to the problem. A custom opaque phone holder was produced using 3D printing (Ultimaker Inc. [https://ultimaker.com]) to control light source quality and quantity during the procedure and for calculating test strip scores (Fig. 2A). This portable phone holder was designed to allow field personnel to conduct experiments and collect results in a convenient and user-friendly manner. Inside the black opaque phone holder, two LED diodes served as a controllable and consistent light source to eliminate noise introduced by any change in light levels (between or during) the test strip reactions, allowing for more accurate and consistent data collection^19^. Frames of the videos (taken using the smart phone positioned in the holder) were captured for different reagents according to the recommended reading time given by the user manual. For test strip RGB value measurement (Fig. 2B), an automated machine learning method, shown in Fig. 2C, was used as a replacement for traditional, manual methods. Our computer vision workflow, named TestStripDX, uses YOLOv4^18^, which is one of the most mature, accurate, and popular computer vision models available, to isolate a target feature in an image and provide the metadata associated with the object (in this case, each test along the strip), such as position within the coordinates of a rectangular box. Sample pictures of strips were manually annotated and used for training the TestStripDX pipeline (Supplementary Data 1): i.e., these images were used to train a custom detector to identify the different reagent tests along a strip and thereafter, to provide the relevant RGB values for each test (Fig. 2C). Figure 2D shows the comparison between data collected using the manual (ImageJ) versus automated (TestStripDX) methods for a partial list of samples and reagent strips (data shown in Table S1). The very close association of the regression line (R^2^ = 0.996) with the artificial diagonal line validates the utility and accuracy of the computer vision method.

### Coral color score measurements

**Figure 3:**
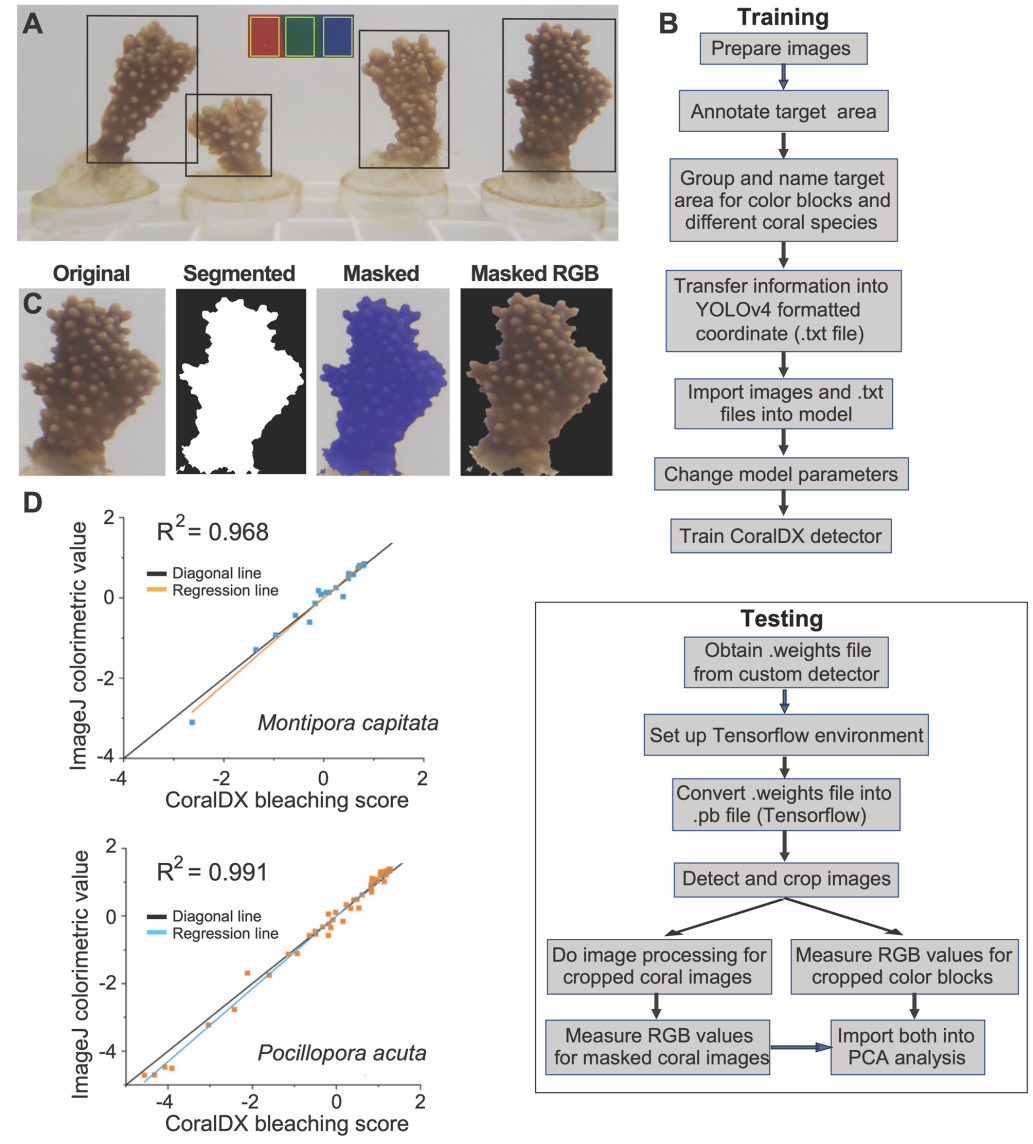
Analysis of coral color scores. (**A**) Example image of *M. capitata* coral nubbins used for automated color score analysis, with target areas marked with the black (coral nubbins) and yellow (color standards) boxes. The nubbin on the far right is used to demonstrate the masking procedure. (**B**) Flow chart for the training and application of the machine learning method (CoralDX) used to analyze coral color scores. (**C**) Example of image processing for one *M. capitata* coral nubbin showing (from left to right) the original, masked, segmented, and masked RGB nubbin, with the final (right) image used for color score measurements. (**D**) Comparison of color score values produced by CoralDX and ImageJ, for a set of representative coral nubbins from the 2019 bleaching experiment (Tables S2, S3). The enforced diagonal matches very closely to the regression line in both analyses.

As described above, color scores provide a proxy for coral health, and are generated by measuring the RGB channels of the bleached (unhealthy) and brown (healthy: i.e., pigmentation provided by the algal symbionts) areas of coral nubbins. For the CoralDX workflow, we trained a custom detector that can recognize coral nubbins, as well as red, green, and blue colored blocks (standards) which are used to normalize the R, G, and B channels in each image (see Supplementary Data 2). The images taken in this case were from a lab environment (Fig. 3A), but this approach could potentially be used in any location provided a background with a uniform color is used in the image. To achieve this goal, a background panel containing the red, blue, and green color blocks would be placed behind (smaller) coral nubbins to make the measurements. It is clear that for large colonies, this approach may prove challenging to apply but we expect that additional testing and modification to the method will allow us to design a better-suited tool for field use. To achieve our goal of automatically obtaining colorimetric measurements of coral nubbins, we needed to devise a method that provides YOLOv4 with areas that are not limited by the standard rectangular boxes used as input for this model. To accommodate irregular nubbin shapes, an additional step (training and testing steps are shown in Fig. 3B) was added to the automated method (Fig. 2C). Our approach uses computer vision-based edge detection to eliminate most of the background surrounding the edges of the coral nubbins, allowing for accurate quantification of the RGB values of the targeted piece of coral. After edge detection, we obtain a picture with a black background highlighting the coral shape as a “mask” (Fig. 3C; 2nd image from left). We then place the mask onto the original coral image, measuring non-zero R, G, and B values (Fig. 3C; 3rd and 4th images from left). This method is superior to selecting coral areas for manual analysis using tools such as the handsfree selection function in ImageJ, which are generally hard to manipulate, have low fault tolerance, and are time-consuming. The RGB values extracted from the coral nubbins and color blocks were investigated using principal component analysis (PCA) to generate Euclidean distances (color scores) among coral nubbins according to treatment, time point, and colony^11,16^. In Fig. 3D, the correlation between bleaching scores generated using CoralDX and ImageJ for *M. capitata* and *P. acuta* are presented for a representative set of nubbins cultured under ambient or thermal stress conditions in the 2019 bleaching experiment (Tables S2, S3). As is apparent, the regression lines show strong correlations (R^2^ = 0.968 for *M. capitata* and R^2^ = 0.991 for *P. acuta*), supporting the utility of the automated method. The data in both cases are very closely associated with the artificial diagonal line shown in the images, substantiating the strong positive correlation between the scores from the two approaches. The small differences in score values between the methods is primarily explained by difficulties in cropping the edges of coral nubbins that are heavily bleached; the contrast between the coral nubbin and the white background is lessened, creating discrepancies between the nubbin edges identified by CoralDX and ImageJ. This issue can be addressed by testing different color backgrounds to find the optimal set-up. Another potential contributing factor is the differences in the area selected for the color blocks between manual (ImageJ) versus automated methods, although this might only have a minor effect on the results. The CoralDX workflow is not computationally intensive and easily portable to other platforms, such as personal computers and smart phones, allowing for easy deployment in the field.

### Test strip results

**Figure 4:**
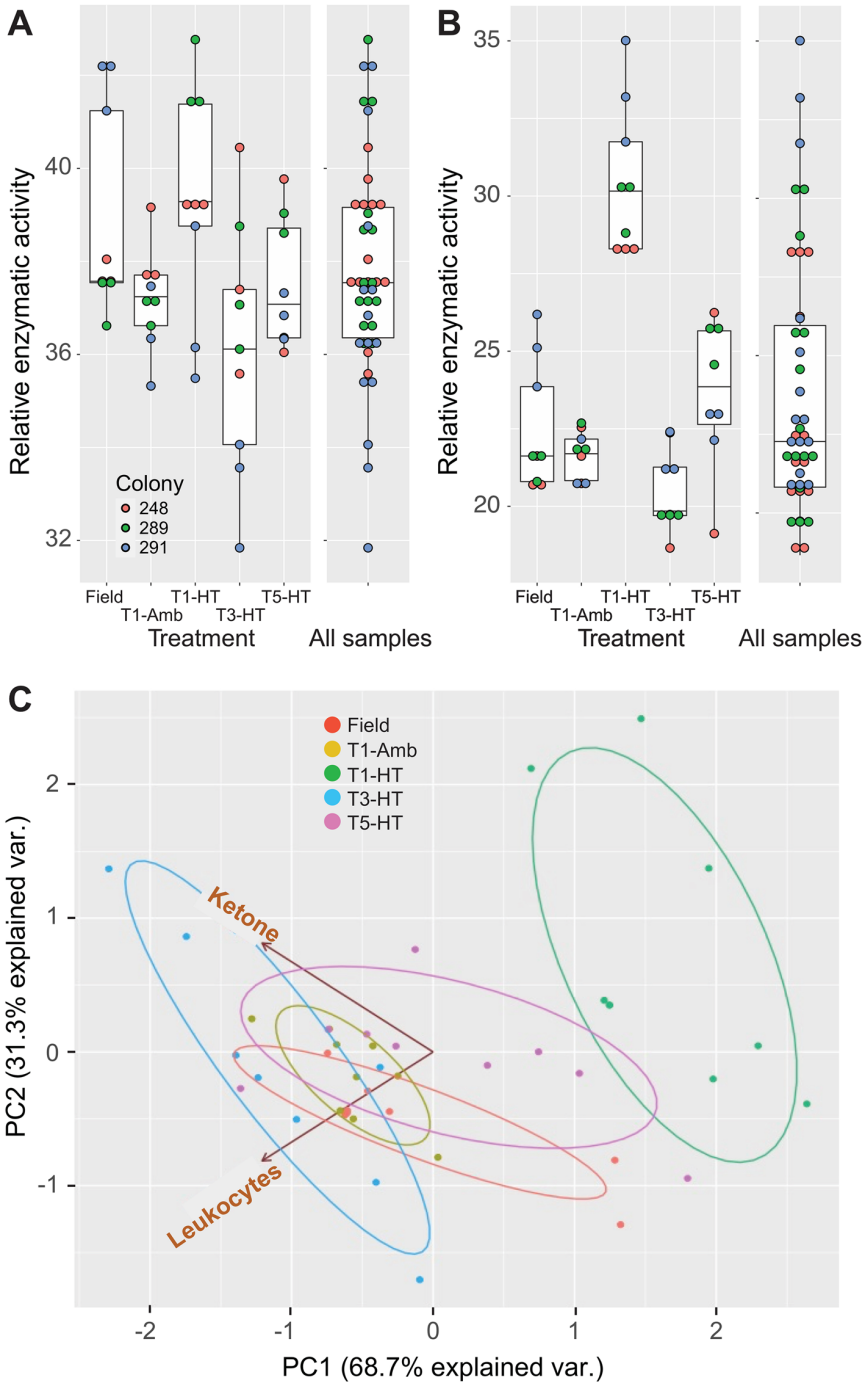
Test strip results from the 2019 bleaching experiment. (**A**) Ketone test strip results for *M. capitata*, showing genotype-specific (see legend) differences in response. (**B**) Leukocytes test strip results for *M. capitata*, showing genotype-specific (see legend) differences in response. These are standard box plots, with the boxes representing the first (Q1) to third (Q3) quartiles. The lines in the boxes are the median (Q2) values and lines (“whiskers”) extending beyond the boxes are the minimum and maximum values, excluding outliers. (**C**) PCA of the ketone and leukocytes test strip data for the field, ambient, and T1-HT, T3-HT, and T5-HT timepoints.

Armed with these new tools, we generated the test strip data for different coral samples, where larger “relative enzymatic activity” (REA, measured using the RGB values) values indicate increased reactivity (i.e., increased levels of products targeted by the test). The Accutest URS-14 100 strips test for ketones (using the sodium nitroprusside reaction) measures acetoacetate and assumes the presence of β-hydroxybutyrate and acetone. The former (β-hydroxybutyrate) acts as a signal to regulate metabolism and maintain energy homeostasis during nutrient deprivation. In this process, β-hydroxybutyrate is converted to acetoacetate. Ketone bodies are transported into tissues and converted into acetyl-CoA by thiolases, which then enters the TCA cycle and is oxidized in the mitochondria for energy. Bleaching in corals which are incapable of obtaining adequate energy stores through heterotrophy results in diminished growth rates, degraded reproductive capacity, amplified susceptibility to disease, and elevated mortality rates for the entire colony^20^. Although ketosis has not been explored in cnidarians, transcriptomic data generated from the *M. capitata* samples measured in this study^12^ demonstrate expression of the KEGG pathway for degradation of ketone bodies (Fig. S1). The combination of time-point and treatment (field, T1-AT, T1-HT, T3-HT, T5-HT) was the most significant factor impacting the ketone REA scores (*p*-value = 0.010) (see details of the PERMANOVA analysis in the “Methods” section). Given this framework, we find that the *M. capitata* samples remain steady throughout the bleaching period, except for a decrease in enzymatic activity at T3-HT (Fig. 4A). This result is supported by the transcriptomic data, which shows this pathway to be uniformly expressed at all timepoints, except T3-HT, when acetyl-CoA C-acetyltransferase is up-regulated in comparison to T1-HT (fold change [FC] = 1.52) and down-regulated at T5-HT (FC = − 1.84) (Table S4). A possible explanation for this result is that at T3-HT, when the first evidence of bleaching was present, the photosynthetic rate of the symbiotic algae was elevated due to the thermal stress, resulting in greater energy production and a decrease in the abundance of ketone bodies within the coral, but without sufficient stress to cause significant bleaching. During this time, acetyl-CoA C-acetyltransferase enzymatic activity favored the production of acetoacetyl-CoA. However, as dysbiosis continued and the corals no longer had access to the photosynthetic products provided by the symbionts, they produced more acetoacetate and ketosis was detected by the test strips. This response occurs despite the observation that *M. capitata* can persist heterotrophically and meet much of its energy needs in the absence of algal symbionts^21^. Interestingly, the three *M. capitata* field samples show similar amounts of ketone bodies to lab stressed corals, suggesting the presence of stressors in the natural environment.

The leukocyte test measures the activity of leukocyte esterase (presence of white blood cells) and other signs of infection in human subjects. This test putatively assesses the coral innate immunity response, which includes the same phases in response to infection and loss of tissue integrity as other invertebrates: recognition, signaling, and effector response^22^. Corals contain multiple types of immune cells, such as amoebocytes and fibroblasts^23^. Amoebocytes are amoeboid cells residing in the mesoglea that remove necrotic tissue, encapsulate foreign particles, and generally display phagocytic activity to aid in organism defense against pathogens, which is the cnidarian principal mechanism of immunity^24^. Amoebocytes can be melanin-containing, agranular, or granular based cells, depending on the signaling pathway^22^. *M. capitata* shows an overall increase in enzymatic activity at T1-HT, signaling a heightened immune response (Fig. 4B). This relatively higher level of enzymatic activity decreases at T3 and T5. The field samples show levels that are comparable to the T3-HT and T5-HT thermal stress acclimated colonies and the T1 ambient (Amb) colonies. Again, a combination of time-point and treatment was the most significant factor impacting the leukocyte REA scores (*p*-value = 0.001). Additional research needs to be done to understand the cause of this response. Nonetheless, the results are consistent with the widely accepted hypothesis that *M. capitata* adapts well to bleaching conditions^12^. PCA of the *M. capitata* test strip results shows a clear separation of the T1-HT coral data from the T3-HT and T5-HT values, with the ambient and field samples intermixed among the latter two timepoints (Fig. 4C). This result again highlights the initial robust response to stress by *M. capitata* followed by acclimation to the heat treatment that is reflected in the field samples.

A noteworthy aspect of the test strip results is the divergence in response to thermal stress among different colonies. This result has also been found for coral metabolomic data^11^. As described above, each holobiont integrates a complex set of biotic interactions between the host animal and microbiome, explaining the high variation in ketone and leukocytes test results, often between replicate nubbins from one colony and more frequently, between different colonies (Fig. 4A). Existing data using omics methods demonstrate that the stress response of the coral holobiont varies from colony to colony^15^. The metabolome is controlled by the coral animal genotype, microbial consortium, and environmental conditions, among other factors, and can fluctuate greatly based on individual metabolite turnover rates and the timing of sampling^25,26^. Therefore, accounting for natural variation in the stress response phenotype and its importance for effective testing methods is a crucial aspect of our work. Our results demonstrate that broad population level sampling (dozens to 100 s of colonies/genotypes) is likely needed to account for the inherent genetic and metabolic variation present in wild coral populations.

**Figure 5:**
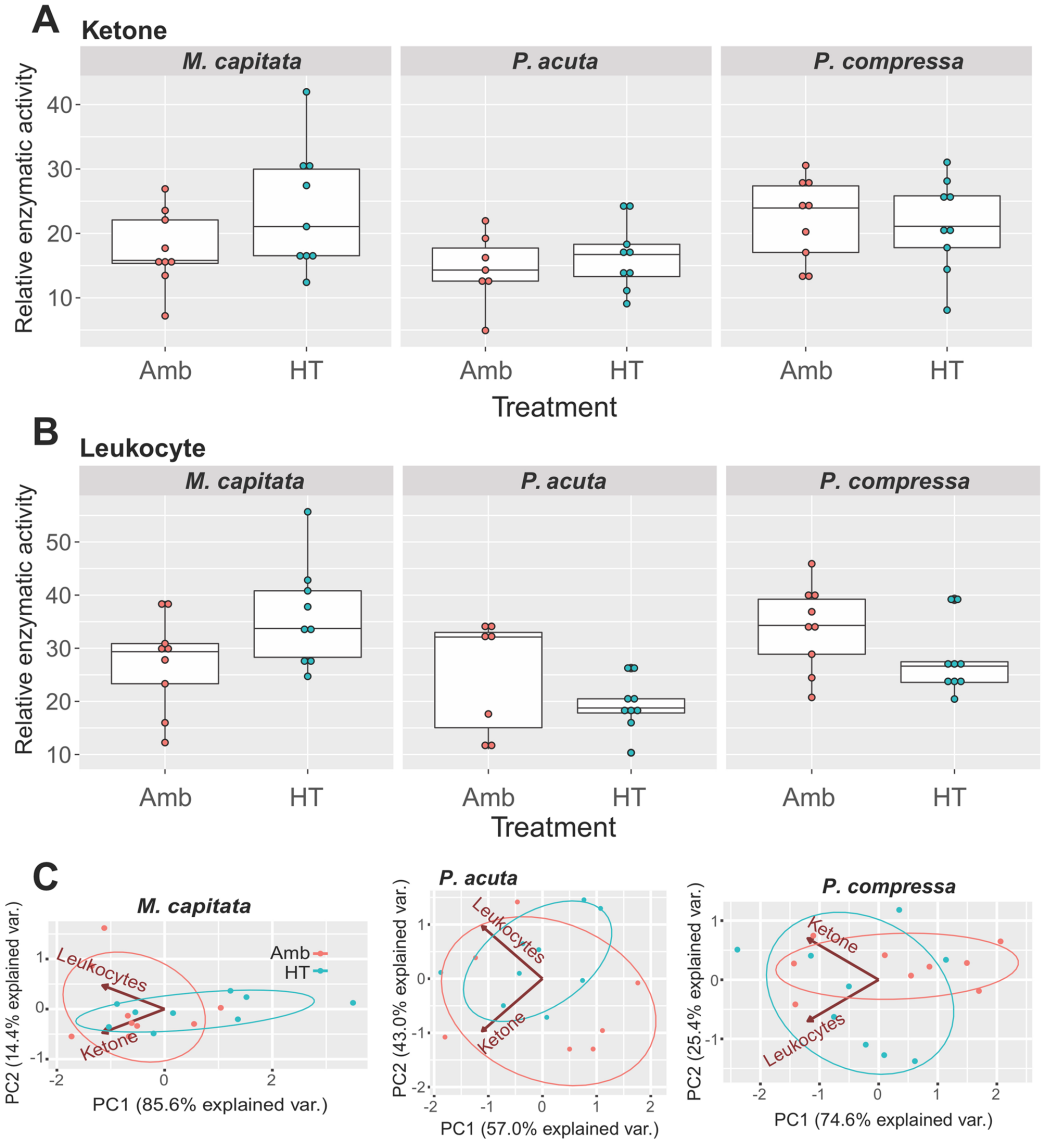
Test strip results from the 2021 bleaching experiment for three Hawaiian coral species. (**A**) Ketone test strip results for *M. capitata*, *P. acuta*, and *P. compressa* showing genotype-specific variation in response under the ambient (Amb) and high temperature (HT) treatments. (**B**) Leukocytes test strip results for *M. capitata*, *P. acuta*, and *P. compressa* showing genotype-specific variation in response under the ambient and high temperature (HT) treatments. These are standard box plots, with the boxes representing the first (Q1) to third (Q3) quartiles. The lines in the boxes are the median (Q2) values and lines (“whiskers”) extending beyond the boxes are the minimum and maximum values, excluding outliers. (**C**) PCA of the ketone and leukocytes test strip data for the Amb and HT treatments for the three Hawaiian coral species.

We also did a more limited analysis of coral stress responses using the ketone and leukocyte test strips with three species (*M. capitata*, *P. acuta*, *P. compressa*) in a 2021 bleaching experiment in which we sampled multiple coral genotypes at time 0 and after 9 days of heat treatment (3ºC increase from ambient; see Methods). These results are based on analysis of 8–9 different coral genotypes (summarized in Fig. 5). Interpreted in the same way as described above, we see that there is substantial genotype-based variation in the stress response. Nonetheless, consistent with the 2019 data, *M. capitata* shows evidence of a thermal stress response in the ketone and leukocytes tests (Fig. 5A, B). *P. acuta* shows a more limited response, whereas *P. compressa* appears to have fully acclimated to the stress regime with lowered reactivity at the end of the experiment. Species identity was the most significant factor impacting the leukocyte and ketone REA scores (*p*-value = 0.002 and 0.033, respectively), but a combination of species and treatment (AT vs. HT) was found to be significant for leukocytes (*p*-value = 0.026). PCA of the *M. capitata* test results shows separation between the ambient samples and some of the high temperature treated samples along PC1; both the ketone and leukocytes tests contribute to the spread of samples along PC1. This reinforces the conclusion that there is evidence for a thermal stress response in the ketone and leukocytes tests of *M. capitata* however, the significant genotypic variability (particularly apparent when compared with Fig. 4) obscures the differences between the conditions for some of the samples. PCA of the *P. acuta* and *P. compressa* test results mirrors our conclusion that the ketone and leukocytes tests in these species show a limited response to stress, with no separation between the samples from the different conditions.

### Analysis of wild populations

**Figure 6:**
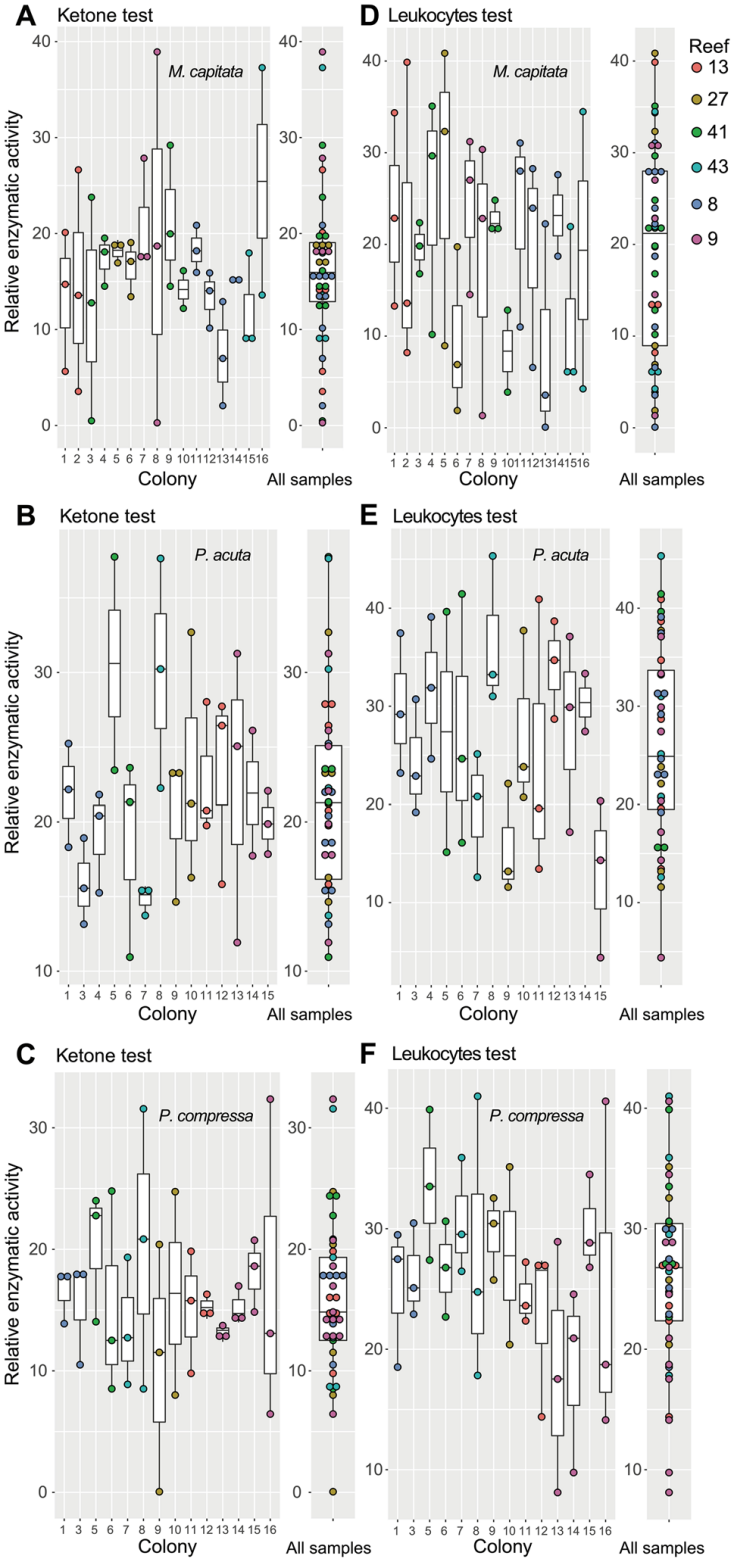
Test strip results from analysis of the 2021 collection of three Hawaiian coral species from the wild. (**A** and **D**) Ketone and leukocytes test strip results, respectively, for *M. capitata *showing intraindividual variation and among the different reefs (see legend) that were sampled (Fig. 1B). (**B** and **E**) Ketone and leukocytes test strip results, respectively, for *P. acuta* showing intraindividual variation and among the different reefs that were sampled. (**C** and **F**) Ketone and leukocytes test strip results, respectively, for *P. compressa* showing intraindividual variation and among the different reefs (see legend) that were sampled. These are standard box plots, with the boxes representing the first (Q1) to third (Q3) quartiles. The lines in the boxes are the median (Q2) values and lines (“whiskers”) extending beyond the boxes are the minimum and maximum values, excluding outliers.

To assess natural variation, we collected apparently healthy *M. capitata*, *P. acuta*, and *P. compressa* nubbins (3 replicate nubbins per colony) from six reefs in Kāneʻohe Bay and from three sites near the Hawaiʻi Institute of Marine Biology on Coconut Island (Moku o Loʻe) (see Fig. 1) and analyzed these tissue extracts using the ketone and leukocytes tests. This analysis shows wide variation in the results with some interesting exceptions. Species identity was the most significant factor impacting the leukocytes and ketone REA scores (*p*-value = 0.002 and 0.001, respectively), but for leukocytes, colony identity, regardless of species, was also found to be a significant factor (*p*-value = 0.040). The *M. capitata* ketone test results are consistent among different reefs and within the same colony with the exception of some colonies (e.g., Colony 8 from Reef 9 and Colony 16 from Reef 43) that show wide intra-colony variation (Fig. 6A). Most of the ketone data for *M. capitata* fall between REAs of 10–20. In contrast, *P. acuta* shows more variation in the wild populations for the ketone test, suggesting that many of these coral colonies live under stressful conditions in the field (Fig. 6B). A similar situation to *M. capitata*, in terms of REAs, holds for the ketone test of *P. compressa* colonies that show more limited variation (Fig. 6C). The leukocytes test shows high variation for *M. capitata* (Fig. 6D), *P. acuta* (Fig. 6E), and *P. compressa* (Fig. 6F) colonies. These results again point out the complex nature of genome-environment interaction with respect to metabolic syndromes, both at the colony level and among different regions (replicates) of the same colony. For example, *P. compressa* Colonies 13–15 from Reef 9 show little to no intra-colony variation for the ketone test, yet another colony from this reef (Colony 16) shows high variation among replicates (Fig. 6C). In contrast, *P. compressa* Colonies 13–15 are far more variable when using the leukocytes test (Fig. 6F). On the basis of the more predictable lab-based results reported above, we interpret these “noisy” field data as evidence of the immense variation in the stress phenome of wild coral populations. Overall, the field results indicate that a starting set of test strip values, followed by repeated field sampling over time of wild colonies may be needed for accurate stress diagnosis, rather than the one-time measurement approach used here. Clearly, more work is needed with wild colony analysis, particularly under varying degrees of thermal stress and apparent bleaching, to fully realize the potential of the technique we present.

### Analysis of transcriptomic data

**Figure 7:**
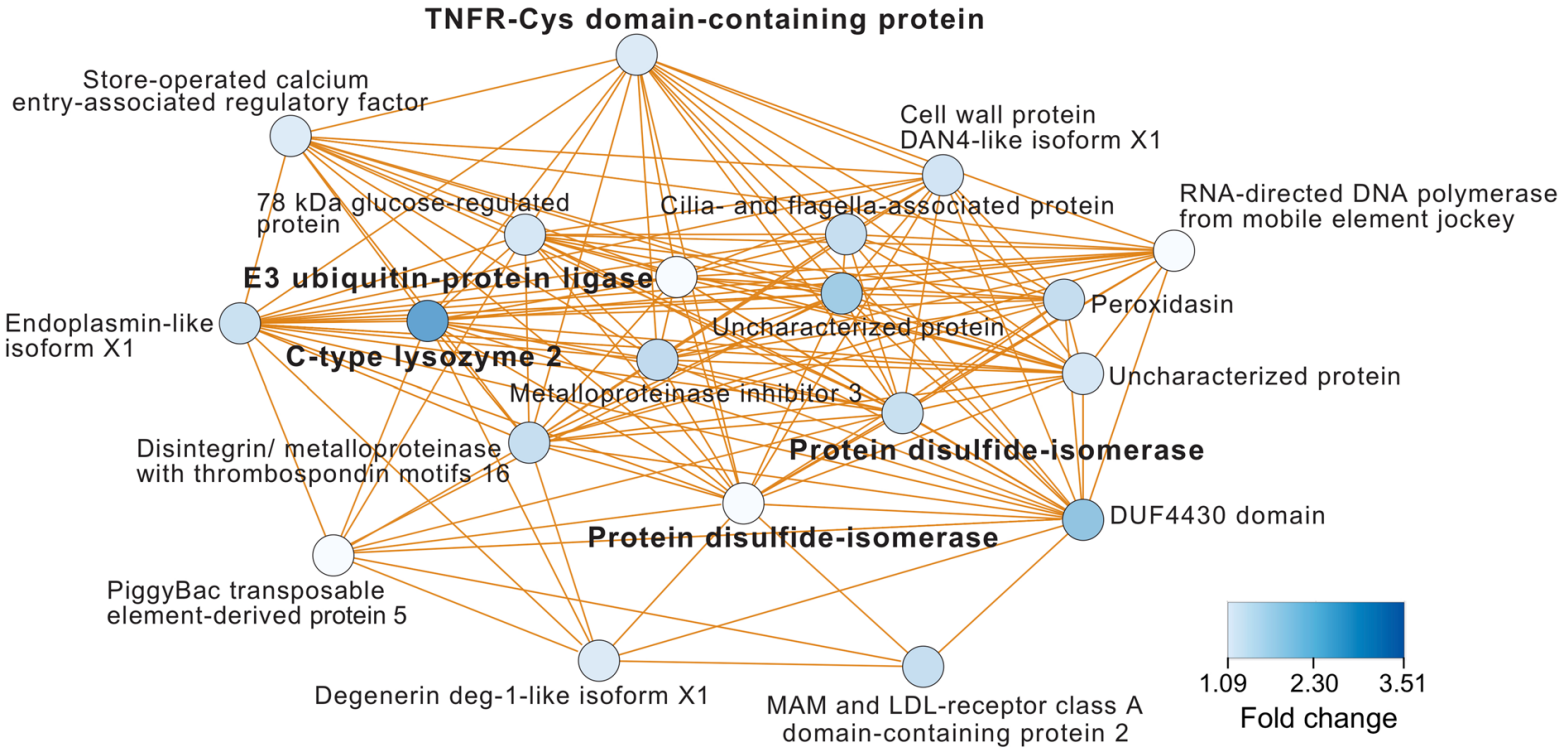
Gene co-expression analysis. Module 2 representing significantly up-regulated genes in *M. capitata* from T1-HT in the 2019 differential gene expression analysis^12^. This module is enriched in genes involved in the wound healing and immune response. The legend for level of up-regulation is shown. Putative gene annotations are also shown.

To identify pathways that may support the *M. capitata* leukocytes test strip results, which showed the most response in terms of change in REA at T1-HT (Fig. 4B), we analyzed existing transcriptomic (RNA-seq) data derived from the same coral nubbins. The RNA-seq and metabolomic data from these samples have been previously analyzed^11,12^. Here we searched for co-expression modules that contain genes that are up-regulated at the start of the thermal stress regime (T1-HT) when compared to the ambient treatment. It is at this timepoint that we find a strong cross-reaction with the leukocytes test, followed by loss of cross-reactivity at T3-HT and T5-HT (back down to T1-Amb levels), putatively indicating acclimation (Fig. 4B). As described above, the wound healing response in corals is complex and the (Urinalysis) leukocytes results need to be interpreted as a syndrome involving multiple pathways of stress and immune response. With these considerations in mind, we identified a module (Module 2; see Williams et al.^12^) of up-regulated genes that contains several markers associated with the coral stress response (Fig. 7). These include a tumor necrosis factor-activated receptor (TNFR)-Cys domain-containing protein (fold-change [FC] = 1.12) that is a well-known mediator of apoptosis and cell death that is functionally conserved in corals. Some members of the TNF family are associated with bleaching^27^. The most highly up-regulated gene in this module is C-type lysozyme 2 (FC = 2.42) that provides an anti-microbial function (e.g., digestion of peptidoglycan), and is likely expressed as a result of stress-induced dysbiosis in *M. capitata*. Other markers of stress that are up-regulated in Module 2 include E3 ubiquitin-protein ligase (FC = 1.03) involved in protein degradation, two protein disulfide-isomerase (FCs = 1.32, 1.09) involved in cellular defense against protein misfolding via chaperone activity^28^, and a metalloproteinase inhibitor 3 (FC = 1.42) which likely functions as a physiological anti-inflammatory molecule^29^. These data, although not directly substantiating the leukocytes test strip results, provide evidence that the wound healing and immune response pathways were up-regulated in Module 2 at T1-HT (albeit weakly, due to the stress resilience of *M. capitata*) as suggested by Fig. 4B.

## Summary

This work lays the foundation for targeting conserved animal, or potentially coral-specific pathways and exploiting existing, commercially available tools to diagnose holobiont health. Although clearly in need of further development and more corroborative data (e.g., PAM fluorometry to measure algal symbiont photosynthetic efficiency, stress protein expression levels), we see great potential for the aforementioned approaches. Any field method for assessing coral health will need to be inexpensive, easy-to-use, and rapidly deployable. Test strips fulfill these needs, as they have for many years in human health monitoring. This approach will however be strengthened when test strips are designed for well-characterized coral stress metabolites (e.g., dipeptides or sex steroids) that measure physiological states in these species (work in progress). We see this as a logical next step in the approaches described herein. Given that the needed technical improvements can be made, there still remains the major question of high genotype-environment interactions among different colonies and even within individual colonies, that will likely make coral stress monitoring very challenging. For this reason, we advocate the use of multiple different rapid tests that address different aspects of coral health to arrive at an index of colony health/stress that is generated and tested using broad surveys of different species in different reefs and regions.

## Materials and methods

### Thermal stress experiment

The 2019 thermal stress experiment was previously described in Williams et al.^8,9^. Briefly, four colonies of both *M. capitata* and *P. acuta* were collected from Kāneʻohe Bay, HI under SAP 2019-60. These two species were chosen because *M. capitata* resists bleaching, whereas *P. acuta* is more bleaching-susceptible under the same levels of thermal stress^30^. Each colony was fragmented into 30 nubbins and cocultured in six tanks: three high temperature tanks (HT) and three tanks containing ambient temperature (Amb) seawater. All tanks utilized flow-through seawater coming directly from Kaneohe Bay. Each nubbin was acclimated to the tank for five days before temperatures in the experimental tanks were slowly increased by 0.4 °C every two days for a total of nine days. The final experimental temperature fluctuated between 30.5 and 31 °C, and was held for approximately 16 days, during which time three timepoints were collected (n = 3 for each genotype). T1 was collected the first day after temperature ramp up ended (22 May 2019), T3 was collected when the bleaching was first detected (3 June 2019), and T5 was the final timepoint collected at the end of the experiment (7 June 2019). Each sample was placed in a Whirlpak, flash frozen in liquid nitrogen, and stored at − 80 °C until analysis. Color scores were collected for each sample throughout the experiment, as explained below.

In the 2021 bleaching experiment, samples were collected from colonies of *M. capitata*, *P. acuta*, and *P. compressa* in Kāneʻohe Bay, HI under SAP 2021-41. Each collected colony was fragmented into six nubbins and distributed across three ambient temperature (Amb) and three high temperature (HT) tanks containing sea water. All tanks utilized flow-through seawater coming directly from Kāneʻohe Bay. Nubbins were left to acclimate to tank conditions for 5 days before temperatures in the high temperature tanks were increased to the final temperature set point. The final experimental temperature fluctuated between 30 and 31 °C, and was held for approximately 9 days. Concluding the stress period, each sample was placed in a Whirlpak, flash frozen in liquid nitrogen, and stored at − 80 °C until processing.

### Wild sample collection

Field nubbins from 16 colonies (n = 3) of *M. capitata*, *P. acuta*, and *P. compressa* were collected from Kāneʻohe Bay during June 2021 under SAP 2021–41. Nubbins from two or more colonies were collected from six reefs [Reef 8, 9, 13, 27, 41, 43 and from three sites near the Hawaiʻi Institute of Marine Biology (Fig. 1B)]. Nubbins were placed in Whirlpaks, drained of excess seawater, frozen in a charged dry shipper, and stored at − 80 °C prior to processing.

### Sample processing, strip application, and strip measurements

Approximately 0.2 g of coral tissue from each nubbin was added to 0.5 mL of Tris HCl (pH 8) buffer in a pre-filled tube with 100-micron silica beads, and then vortexed for one minute. These samples were centrifuged for 5 min before dipping the Accutest URS-10 Urine Reagent Strip in the sample, ensuring that each enzymatic site was saturated, but did not bleed into neighboring tests. For field application, we expect to be able to complete all of the needed processes using manual (e.g., centrifuge) or battery powered equipment. Excess reagent was removed from the strip by gentle tapping so that light reflections from the buffer did not alter RGB values from the enzymatic test. A test strip was measured with only the pure buffer (i.e., a blank), to control for the baseline buffer reactivity in each enzymatic test. Experimental test strip RGB values were subtracted from the respective blank value and referred to as “relative enzymatic activity” (REA). As values increase, this corresponds to increased reactivity of the given sample. PERMANOVA analysis of the REA values was carried out in R, using the vegan^31^ package (permutations = 999, method = "bray").

### Test strips and color score analysis

To train the YOLOv4 model used in the test strip analysis workflow (TestStripDX; shown in Fig. 2B), we analyzed 46 test strips (Supplementary Data 1) that were manually labeled (using Roboflow [https://roboflow.com]; Annotate—Roboflow) with the positions of each test along the strip (i.e., each test was represented/encapsulated by an ‘information area’); each area was labeled according to what type of test it captured (i.e., the “group information” of each information area). These annotations were transformed into coordinates and used to train the YOLOv4 detector. The target areas extracted from each image by the trained YOLOv4 detector (i.e., the 10 tests on each strip) are imported into the colorimetric measurement software in MATLAB (https://www.mathworks.com/) to generate the REA values produced by the TestStripDX workflow.

To train the YOLOv4 model used in the color score analysis workflow (CoralDX; shown in Fig. 3B), we analyzed 40 images (Supplementary Data 2) that were manually labeled (using Roboflow) with the positions of each coral nubbin and the colored blocks used as standards for normalization; each area was labeled accordingly, with the annotations transformed into coordinates and used to train the YOLOv4 detector. The 40 images used to train YOLOv4 were selected because they are of nubbins collected at different time points in the thermal stress cycle. In the CoralDX workflow, the RGB values of the areas covering the colored blocks extracted from each image by the trained YOLOv4 detector are used for downstream color score analysis. The areas covering the coral nubbins that are extracted from each image are further processed by Tensorflow (https://www.tensorflow.org), which is an open-source software library for machine learning and artificial intelligence, to detect the edges of each coral nubbin. The custom detectors were trained in Google colab with a clone of the YOLOv4 darknet from Github (https://www.github.com/AlexeyAB/darknet), implemented with the NVDIA CUDA GPU acceleration toolkit. After training, for both workflows, we obtained a weights file which includes all information about the custom detector and can predict the target areas with 99–100% accuracy. To utilize the weights files/custom detector in a convenient manner on desktop computers, we created the Tensorflow GPU environment using the CUDA toolkit and the Anaconda environment (https://www.anaconda.com) for processing graphical input. To implement YOLOv4 using TensorFlow, we first converted the weights into the corresponding TensorFlow model files and then ran the custom models on desktop computers. We used computer terminal commands to crop the detected areas and saved these as new images for subsequent steps in image processing.

### Gene co-expression gene networks

To identify pathways that may support the *M. capitata* leukocytes test strip results, which provided the strongest signal, we analyzed existing transcriptomic (RNA-seq) data derived from the same coral nubbins. The RNA-seq and metabolomic data from these samples are presented and analyzed in Williams et al.^11,12^, where all experimental details are available. Briefly, to visualize these data, we created gene co-expression networks using the R-package DGCA^32^ to determine the correlation between pairs of genes respectively for each sampled time point. Pairwise correlations were calculated with the function matCorr using the Pearson method and the functions matCorSig and adjustPVals were used to calculate and adjust (with the Benjamini–Hochberg method) the correlation *p*-values, respectively. We only considered pairs with an adjusted *p*-value ≤ 0.05 to construct the networks. Module detection was done using the functions hclust (method = “average”) and cutreeDynamicTree (minModuleSize = 10 and deepSplit = TRUE). Modules were labeled manually based on NCBI database searches.

## Supplementary Information

Supplementary Information 1.

Supplementary Information 2.

Supplementary Information 3.

Supplementary Information 4.

Supplementary Information 5.

Supplementary Information 6.

Supplementary Information 7.

## Data Availability

The *Montipora capitata* transcriptome data are available under NCBI BioProject ID: PRJNA694677. The TestStripDX workflow is available from https://github.com/dbsymbiosis/TestStripDX and the CoralDX workflow from https://github.com/dbsymbiosis/CoralDX. Codes used for normalization/plotting of test strip results and generating the PCA plots are available from http://github.com/dbsymbiosis/Test-Strip-data-analysis.
