# Supplementary figures and images for: Development of a portable toolkit to diagnose coral thermal stress
Source: Sci Rep. 2022 Aug 24;12:14398. doi: 10.1038/s41598-022-18653-3 (PMC9402530; doi:10.1038/s41598-022-18653-3)

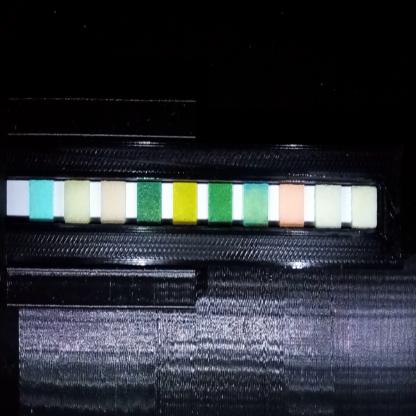

Supplement: Supplementary file 1 — Supplementary Information 1. [file 41598_2022_18653_MOESM1_ESM.zip › Supplementary Data 1/training/3MC206-1721-Tris_0-3g_jpg.rf.f9126aea29c2f4abfac41c51186f704a.jpg]

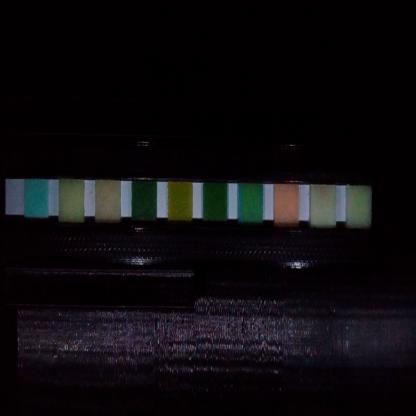

Supplement: Supplementary file 1 — Supplementary Information 1. [file 41598_2022_18653_MOESM1_ESM.zip › Supplementary Data 1/training/3MC248-1331-Tris_0-2g_jpg.rf.51fb8e79b464a6f564131db861ac1904.jpg]

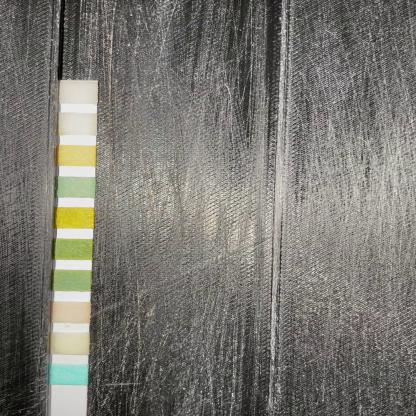

Supplement: Supplementary file 1 — Supplementary Information 1. [file 41598_2022_18653_MOESM1_ESM.zip › Supplementary Data 1/training/capture_123290_jpg.rf.c14548b81a49752a1238a6d5d5014d41.jpg]

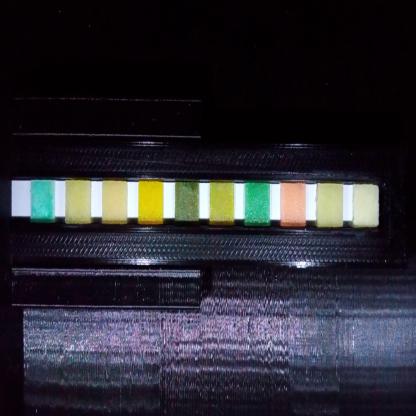

Supplement: Supplementary file 1 — Supplementary Information 1. [file 41598_2022_18653_MOESM1_ESM.zip › Supplementary Data 1/training/1MC289-2393-RLT_0-4g_jpg.rf.e6593896b880aeda859379b1e4724e7c.jpg]

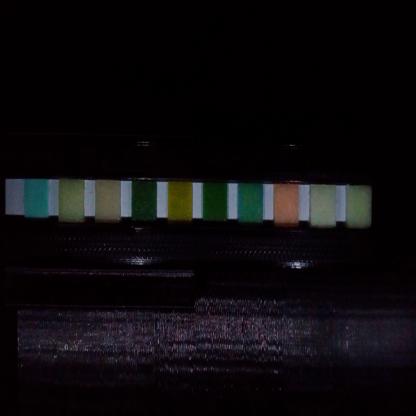

Supplement: Supplementary file 1 — Supplementary Information 1. [file 41598_2022_18653_MOESM1_ESM.zip › Supplementary Data 1/training/1MC248-1331-Tris_0-2g_jpg.rf.a7fcc99f603276f37bd886618d0b6f3a.jpg]

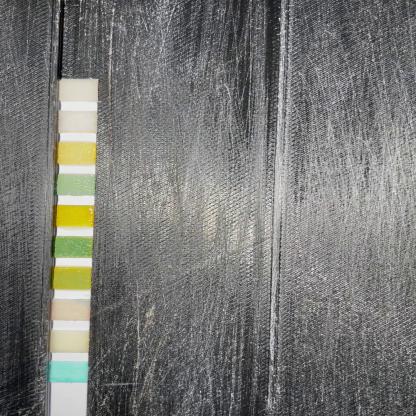

Supplement: Supplementary file 1 — Supplementary Information 1. [file 41598_2022_18653_MOESM1_ESM.zip › Supplementary Data 1/training/capture_254070_jpg.rf.3a68edec87ac6cc891242c0471f68c14.jpg]

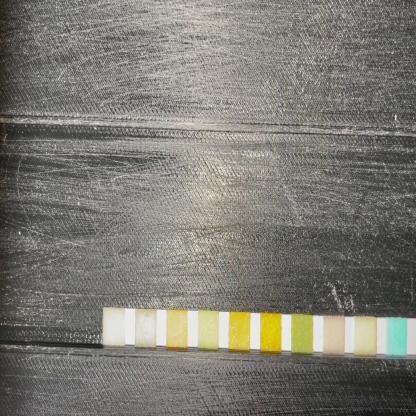

Supplement: Supplementary file 1 — Supplementary Information 1. [file 41598_2022_18653_MOESM1_ESM.zip › Supplementary Data 1/training/capture_1063-CL20_jpg.rf.25952364aa1eb18ab05fc4501c5b14f2.jpg]

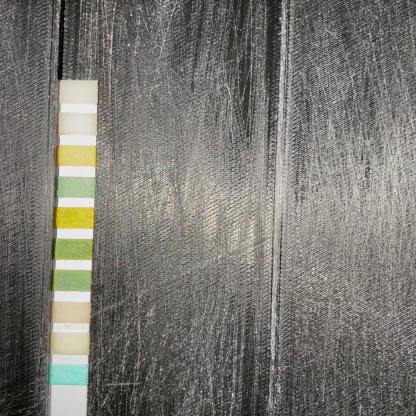

Supplement: Supplementary file 1 — Supplementary Information 1. [file 41598_2022_18653_MOESM1_ESM.zip › Supplementary Data 1/training/capture_1232100_jpg.rf.9568d1b423116c34cf1564b120b60178.jpg]

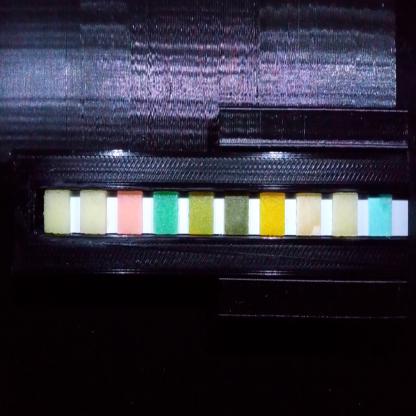

Supplement: Supplementary file 1 — Supplementary Information 1. [file 41598_2022_18653_MOESM1_ESM.zip › Supplementary Data 1/training/1MC248-1331-RLT_0-4g_jpg.rf.dc64e059c18bef13e328bc709c5a3d2f.jpg]

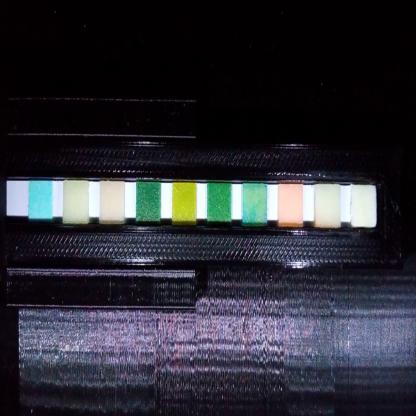

Supplement: Supplementary file 1 — Supplementary Information 1. [file 41598_2022_18653_MOESM1_ESM.zip › Supplementary Data 1/training/3MC206-1721-Tris_0-4g_jpg.rf.7c83fa45533e2ccc94bbe5cf15d2b17a.jpg]

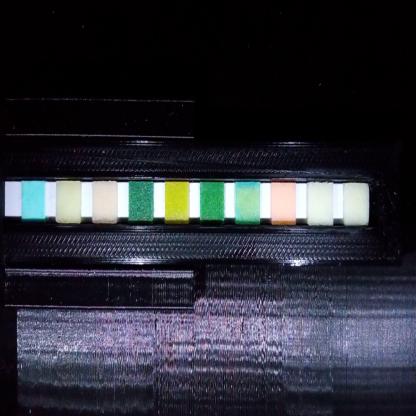

Supplement: Supplementary file 1 — Supplementary Information 1. [file 41598_2022_18653_MOESM1_ESM.zip › Supplementary Data 1/training/3MC206-1721-Tris_0-2g_jpg.rf.235f2d91d22ef8363f31b6c274d23514.jpg]

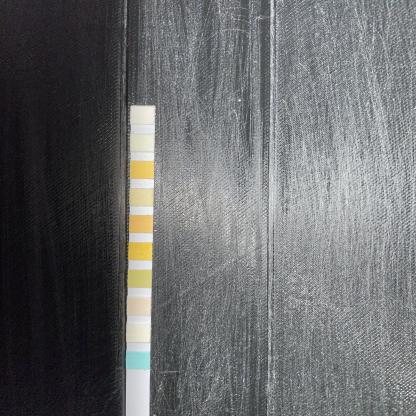

Supplement: Supplementary file 1 — Supplementary Information 1. [file 41598_2022_18653_MOESM1_ESM.zip › Supplementary Data 1/training/blank-strips_bmp.rf.7e5568f5caae590f3368f5544ea1edb9.jpg]

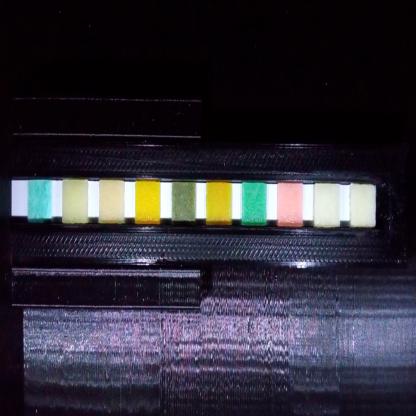

Supplement: Supplementary file 1 — Supplementary Information 1. [file 41598_2022_18653_MOESM1_ESM.zip › Supplementary Data 1/training/1MC289-2393-RLT_0-3g_jpg.rf.946b3ae9c35c88dc18be5640ad15f277.jpg]

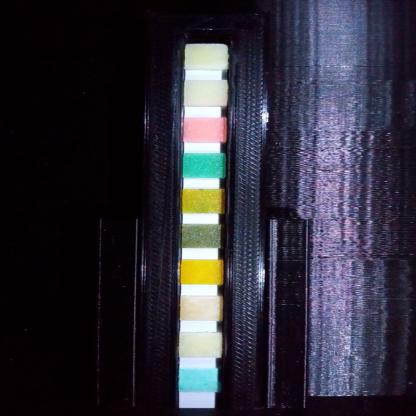

Supplement: Supplementary file 1 — Supplementary Information 1. [file 41598_2022_18653_MOESM1_ESM.zip › Supplementary Data 1/training/1MC248-1331-RLT_0-2g_jpg.rf.339380731982a66f260b9acb4a4b2ff3.jpg]

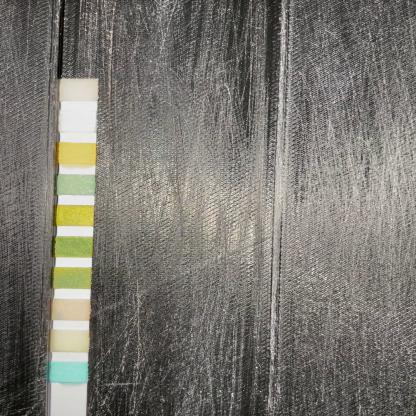

Supplement: Supplementary file 1 — Supplementary Information 1. [file 41598_2022_18653_MOESM1_ESM.zip › Supplementary Data 1/training/capture_122290_jpg.rf.8b3432e4640b6b2fff4c7cf36941ee35.jpg]

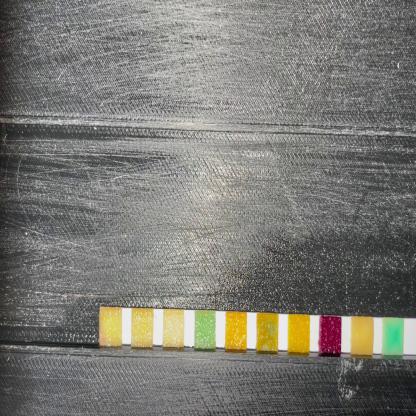

Supplement: Supplementary file 1 — Supplementary Information 1. [file 41598_2022_18653_MOESM1_ESM.zip › Supplementary Data 1/training/capture_1063-rlt100_jpg.rf.c0de2bde0d8a60034d2ba6f434feec24.jpg]

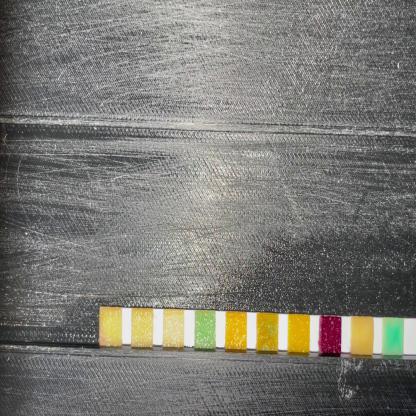

Supplement: Supplementary file 1 — Supplementary Information 1. [file 41598_2022_18653_MOESM1_ESM.zip › Supplementary Data 1/training/capture_1063-rlt70_jpg.rf.9d97fff90c820dd9410ed23f24cec4ef.jpg]

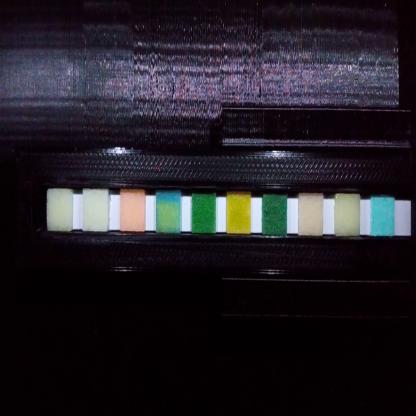

Supplement: Supplementary file 1 — Supplementary Information 1. [file 41598_2022_18653_MOESM1_ESM.zip › Supplementary Data 1/training/3MC248-1331-Tris_0-3g_jpg.rf.ac2c9db8aa8ff7b7b3db4dce8e4b17c2.jpg]

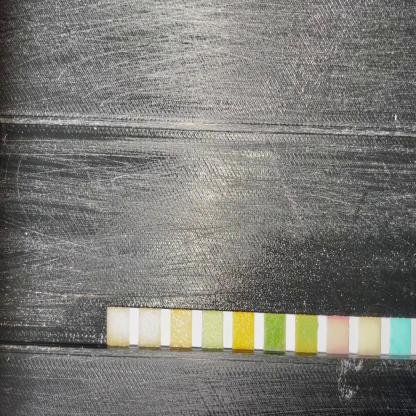

Supplement: Supplementary file 1 — Supplementary Information 1. [file 41598_2022_18653_MOESM1_ESM.zip › Supplementary Data 1/training/capture_1063-EB80_jpg.rf.47c42f1e84391727481452dacd21b657.jpg]

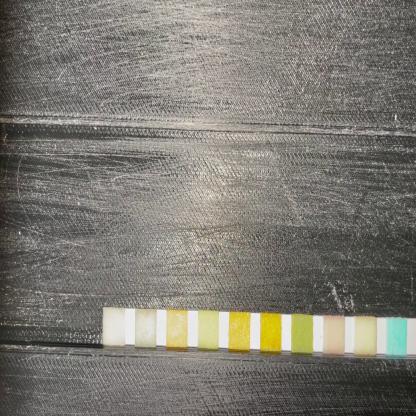

Supplement: Supplementary file 1 — Supplementary Information 1. [file 41598_2022_18653_MOESM1_ESM.zip › Supplementary Data 1/training/capture_1063-CL30_jpg.rf.dd9712268a8a065aebba2335bed4fede.jpg]

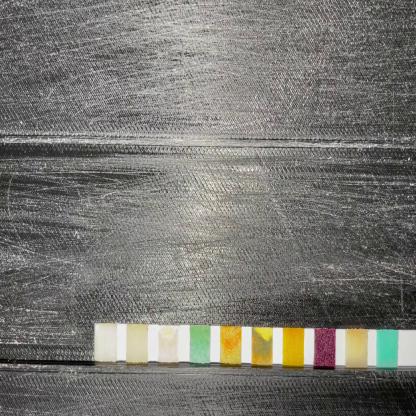

Supplement: Supplementary file 1 — Supplementary Information 1. [file 41598_2022_18653_MOESM1_ESM.zip › Supplementary Data 1/training/capture_1181rlt80_jpg.rf.d036ec9e8763950ca6b793f6c27befea.jpg]

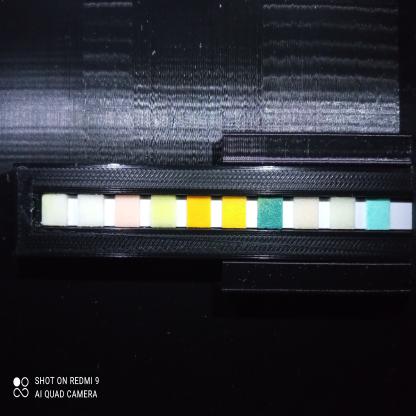

Supplement: Supplementary file 1 — Supplementary Information 1. [file 41598_2022_18653_MOESM1_ESM.zip › Supplementary Data 1/training/1631230556195_jpg.rf.85a90254df54e4e116e4c0d5d65263db.jpg]

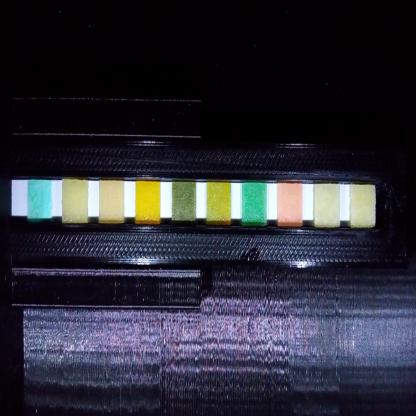

Supplement: Supplementary file 1 — Supplementary Information 1. [file 41598_2022_18653_MOESM1_ESM.zip › Supplementary Data 1/training/1MC289-2393-RLT_0-6g_jpg.rf.f23d540152cafce6bce433f8a187bb38.jpg]

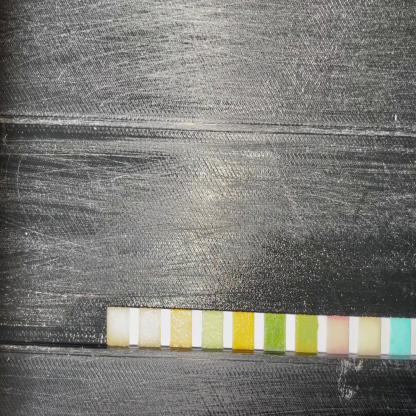

Supplement: Supplementary file 1 — Supplementary Information 1. [file 41598_2022_18653_MOESM1_ESM.zip › Supplementary Data 1/training/capture_1063-EB70_jpg.rf.a61a0f4fa240c1428b69954061dd76d5.jpg]

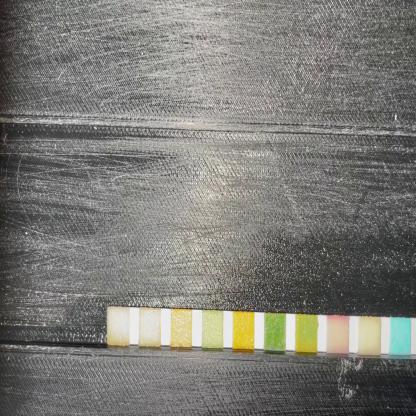

Supplement: Supplementary file 1 — Supplementary Information 1. [file 41598_2022_18653_MOESM1_ESM.zip › Supplementary Data 1/training/capture_1063-EB120_jpg.rf.d35087c7cdae0be6794523689f2ec3e0.jpg]

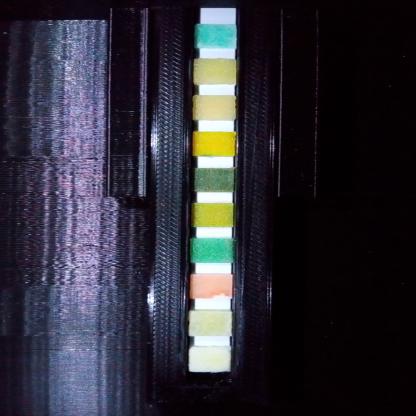

Supplement: Supplementary file 1 — Supplementary Information 1. [file 41598_2022_18653_MOESM1_ESM.zip › Supplementary Data 1/training/3MC248-1331-RLT_0-6g_jpg.rf.32344cc292edea9fb2119f20087b5a92.jpg]

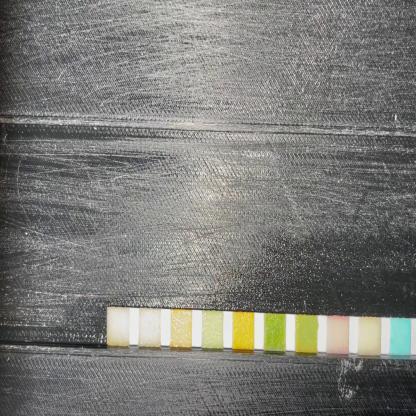

Supplement: Supplementary file 1 — Supplementary Information 1. [file 41598_2022_18653_MOESM1_ESM.zip › Supplementary Data 1/training/capture_1063-EB50_jpg.rf.7e055317f56cdaeb33979d1b48156dda.jpg]

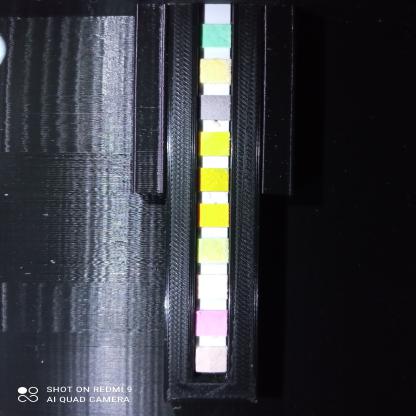

Supplement: Supplementary file 1 — Supplementary Information 1. [file 41598_2022_18653_MOESM1_ESM.zip › Supplementary Data 1/training/1631230556217_jpg.rf.b7c588e826b0292573e6912cf5aad8f3.jpg]

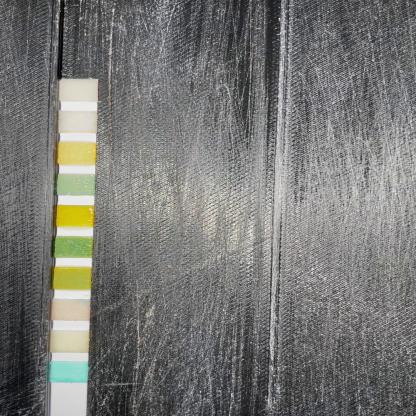

Supplement: Supplementary file 1 — Supplementary Information 1. [file 41598_2022_18653_MOESM1_ESM.zip › Supplementary Data 1/training/capture_254060_jpg.rf.751b73944e5260ae538cfaa4ad5e63fc.jpg]

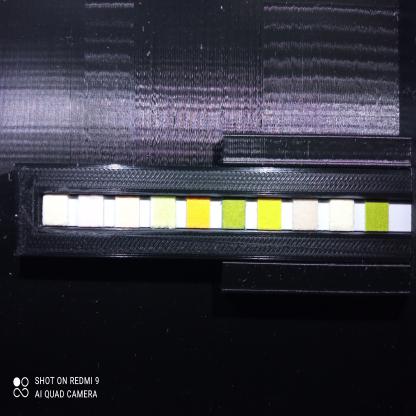

Supplement: Supplementary file 1 — Supplementary Information 1. [file 41598_2022_18653_MOESM1_ESM.zip › Supplementary Data 1/training/1631309273378_jpg.rf.b033f399998ed6b564ae62e9110dba4b.jpg]

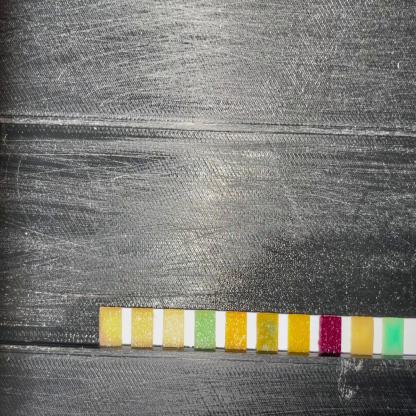

Supplement: Supplementary file 1 — Supplementary Information 1. [file 41598_2022_18653_MOESM1_ESM.zip › Supplementary Data 1/training/capture_1063-rlt120_jpg.rf.aef6232fd1cb447637a4ea8b7813a9b9.jpg]

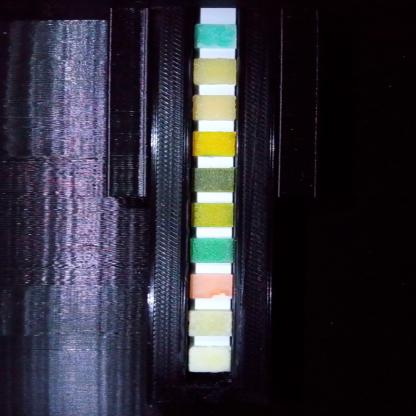

Supplement: Supplementary file 1 — Supplementary Information 1. [file 41598_2022_18653_MOESM1_ESM.zip › Supplementary Data 1/training/1MC248-1331-RLT_0-6g_jpg.rf.cd9678f5f50660de1e7ba85ad57a135f.jpg]

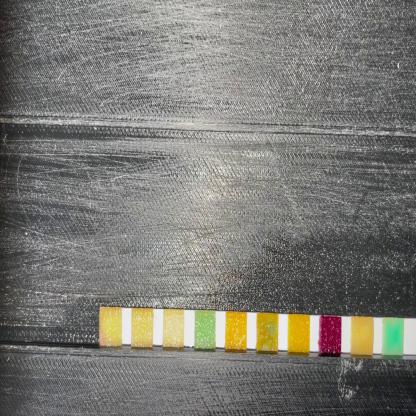

Supplement: Supplementary file 1 — Supplementary Information 1. [file 41598_2022_18653_MOESM1_ESM.zip › Supplementary Data 1/training/capture_1063-rlt110_jpg.rf.801b4d728c2cef5ecbab6de71e7fa789.jpg]

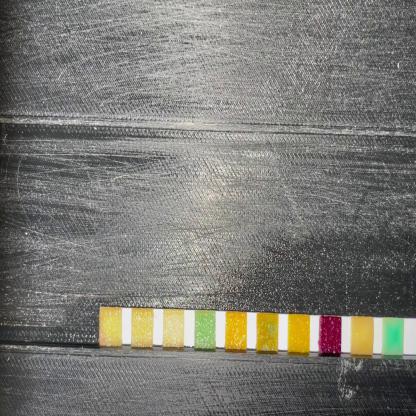

Supplement: Supplementary file 1 — Supplementary Information 1. [file 41598_2022_18653_MOESM1_ESM.zip › Supplementary Data 1/training/capture_1063-rlt90_jpg.rf.113eb122d1edf9f0e8b52fd0a38aebac.jpg]

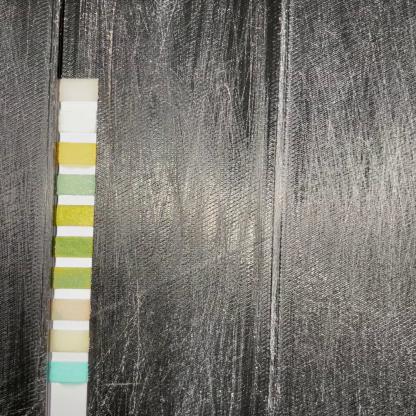

Supplement: Supplementary file 1 — Supplementary Information 1. [file 41598_2022_18653_MOESM1_ESM.zip › Supplementary Data 1/training/capture_122280_jpg.rf.a91c7c3671955cf76e8e92425f61b93b.jpg]

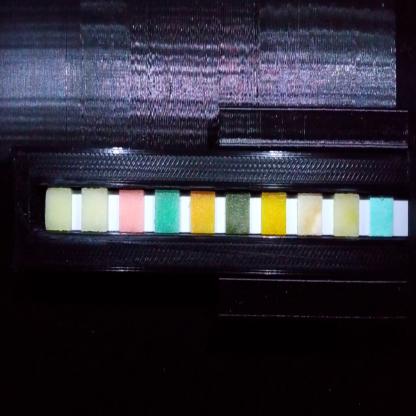

Supplement: Supplementary file 1 — Supplementary Information 1. [file 41598_2022_18653_MOESM1_ESM.zip › Supplementary Data 1/validation/1MC248-1331-RLT_0-3g_jpg.rf.d3af1c039b9bcc7e0a57b7227a17dd2c.jpg]

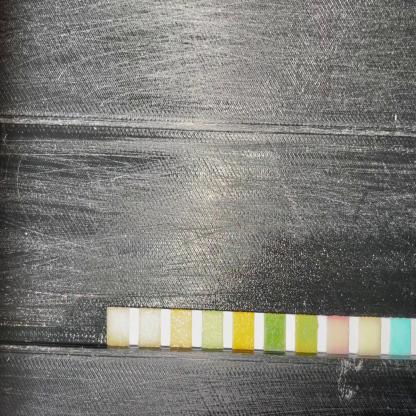

Supplement: Supplementary file 1 — Supplementary Information 1. [file 41598_2022_18653_MOESM1_ESM.zip › Supplementary Data 1/validation/capture_1063-EB90_jpg.rf.920461252a9ae34bad3d0b206ee8e30a.jpg]

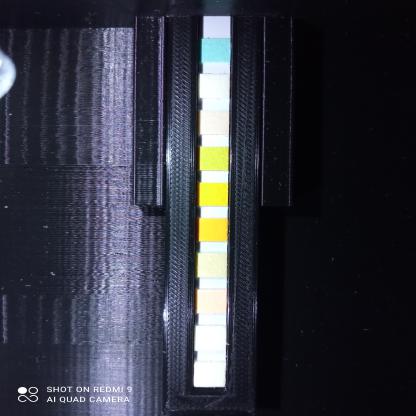

Supplement: Supplementary file 1 — Supplementary Information 1. [file 41598_2022_18653_MOESM1_ESM.zip › Supplementary Data 1/validation/1631230556204_jpg.rf.6d769b04538e7d7d936ccdbfb3193e81.jpg]

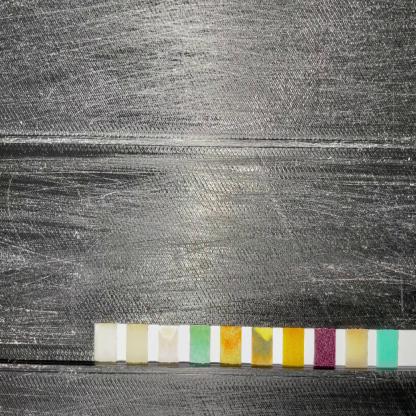

Supplement: Supplementary file 1 — Supplementary Information 1. [file 41598_2022_18653_MOESM1_ESM.zip › Supplementary Data 1/validation/capture_1181rlt90_jpg.rf.8669d33c59c69099d0c002ea11f43f41.jpg]

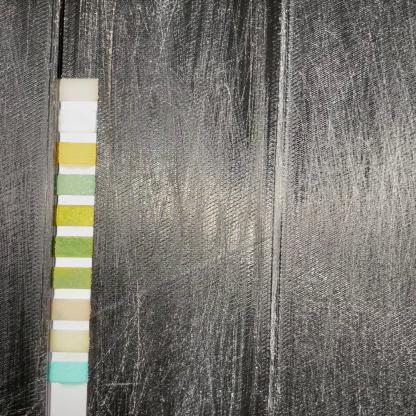

Supplement: Supplementary file 1 — Supplementary Information 1. [file 41598_2022_18653_MOESM1_ESM.zip › Supplementary Data 1/validation/capture_1222100_jpg.rf.cb13676e53e8266d229655eda7644428.jpg]

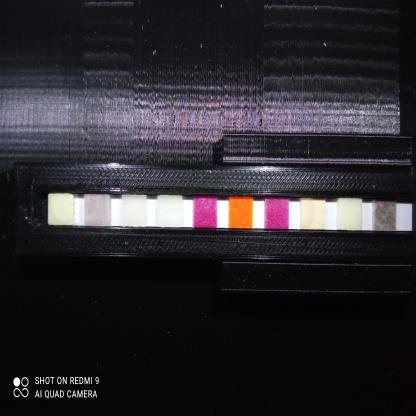

Supplement: Supplementary file 1 — Supplementary Information 1. [file 41598_2022_18653_MOESM1_ESM.zip › Supplementary Data 1/validation/1631230556132_jpg.rf.6dd7874fef8f703bde7cc3bad5b3b066.jpg]

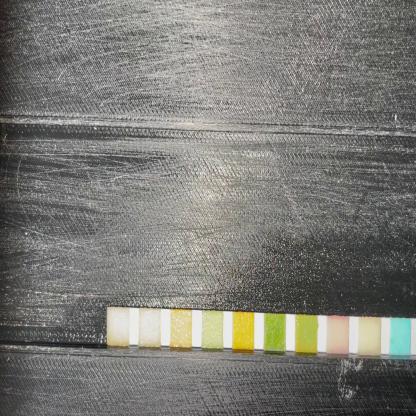

Supplement: Supplementary file 1 — Supplementary Information 1. [file 41598_2022_18653_MOESM1_ESM.zip › Supplementary Data 1/validation/capture_1063-EB60_jpg.rf.6c6296c609b8aee61e495abb3481dab9.jpg]

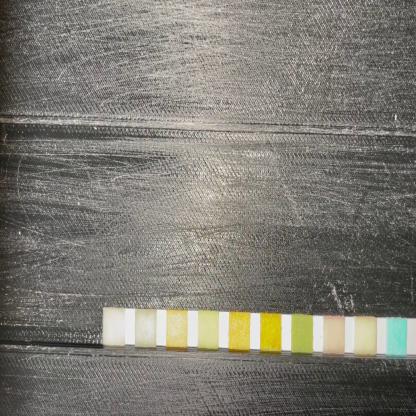

Supplement: Supplementary file 1 — Supplementary Information 1. [file 41598_2022_18653_MOESM1_ESM.zip › Supplementary Data 1/validation/capture_1063-CL40_jpg.rf.150308b17cb7baa7fa646e79bcf7feee.jpg]

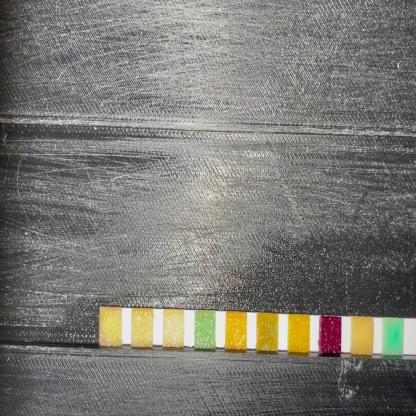

Supplement: Supplementary file 1 — Supplementary Information 1. [file 41598_2022_18653_MOESM1_ESM.zip › Supplementary Data 1/validation/capture_1063-rlt80_jpg.rf.e9cfe3127b1438b0a237840247ac9dad.jpg]

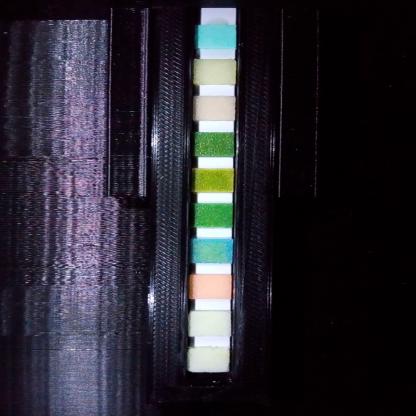

Supplement: Supplementary file 1 — Supplementary Information 1. [file 41598_2022_18653_MOESM1_ESM.zip › Supplementary Data 1/validation/1MC248-1331-Tris_0-6g_jpg.rf.b506f823763094b0495d0bbb101a6c51.jpg]

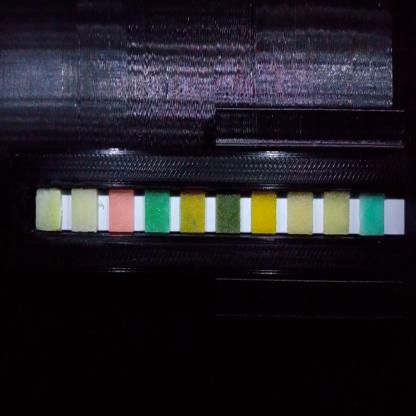

Supplement: Supplementary file 1 — Supplementary Information 1. [file 41598_2022_18653_MOESM1_ESM.zip › Supplementary Data 1/validation/1MC289-2393-RLT_0-2g_jpg.rf.3541158d6d82cf42b056caf23738db57.jpg]

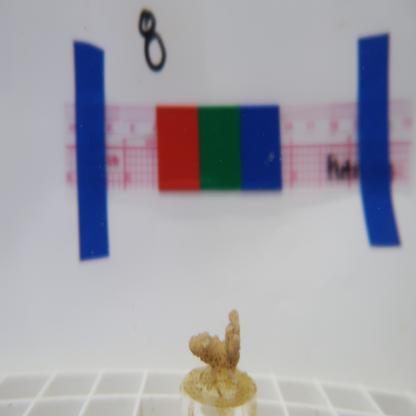

Supplement: Supplementary file 2 — Supplementary Information 2. [file 41598_2022_18653_MOESM2_ESM.zip › Supplementary Data 2/training/Pacuta--5-_jpg.rf.f57f9c7193ac53800ea8019ed8416a34.jpg]

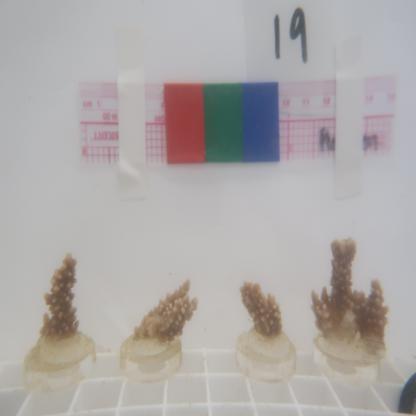

Supplement: Supplementary file 2 — Supplementary Information 2. [file 41598_2022_18653_MOESM2_ESM.zip › Supplementary Data 2/training/Mcap--1-_jpg.rf.61005f9e3a3700a36486332cf9732278.jpg]

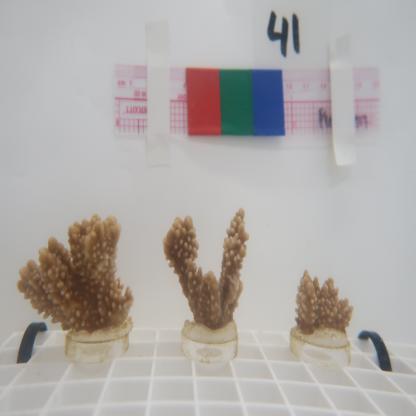

Supplement: Supplementary file 2 — Supplementary Information 2. [file 41598_2022_18653_MOESM2_ESM.zip › Supplementary Data 2/training/Mcap--34-_jpg.rf.6ad9b1c8c591904b19b37d37483a2c9b.jpg]

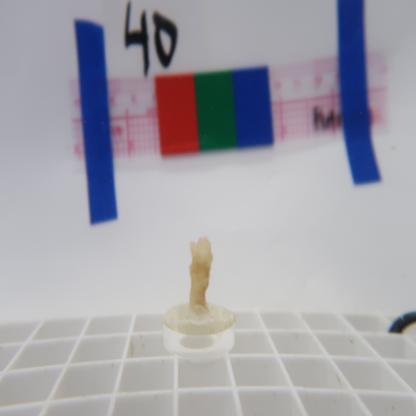

Supplement: Supplementary file 2 — Supplementary Information 2. [file 41598_2022_18653_MOESM2_ESM.zip › Supplementary Data 2/training/Pacuta--22-_jpg.rf.9d431e86bd1d9ba099ac864a2518432c.jpg]

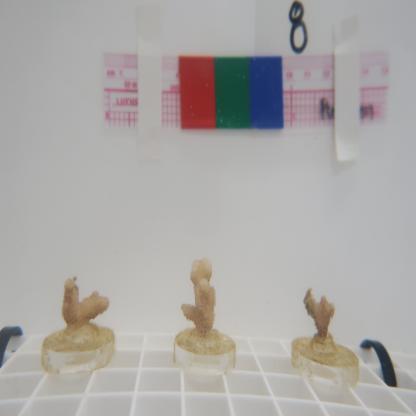

Supplement: Supplementary file 2 — Supplementary Information 2. [file 41598_2022_18653_MOESM2_ESM.zip › Supplementary Data 2/training/Pacuta--25-_jpg.rf.7b133a94f1ab6cc7081f37993a86bc66.jpg]

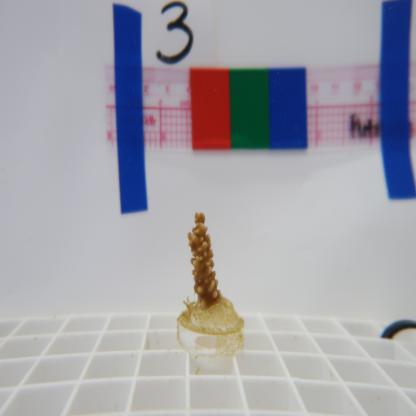

Supplement: Supplementary file 2 — Supplementary Information 2. [file 41598_2022_18653_MOESM2_ESM.zip › Supplementary Data 2/training/CIG_IMG003_jpg.rf.e8fef0a4754027850932f0af963b42d6.jpg]

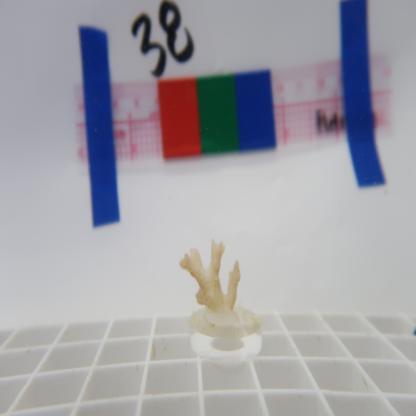

Supplement: Supplementary file 2 — Supplementary Information 2. [file 41598_2022_18653_MOESM2_ESM.zip › Supplementary Data 2/training/Pacuta--21-_jpg.rf.0232fa857bf85e912f59fe205b05b3cf.jpg]

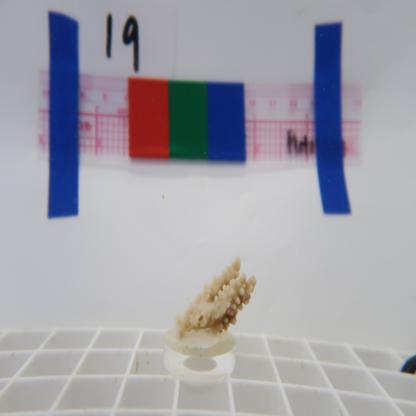

Supplement: Supplementary file 2 — Supplementary Information 2. [file 41598_2022_18653_MOESM2_ESM.zip › Supplementary Data 2/training/CIG_IMG033_jpg.rf.08eba5827b5436aa5a426b57c14c566e.jpg]

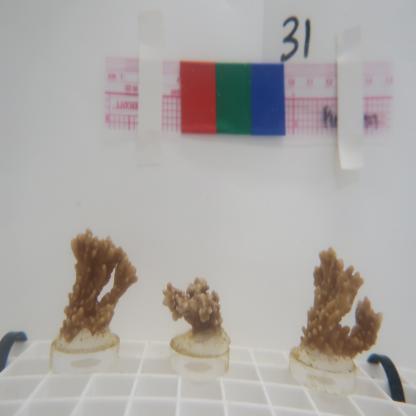

Supplement: Supplementary file 2 — Supplementary Information 2. [file 41598_2022_18653_MOESM2_ESM.zip › Supplementary Data 2/training/Mcap--32-_jpg.rf.4949280e70e29c839216135b76b2f025.jpg]

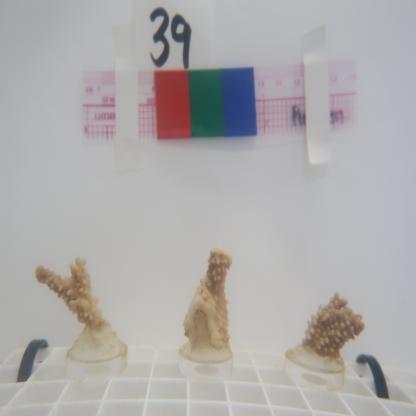

Supplement: Supplementary file 2 — Supplementary Information 2. [file 41598_2022_18653_MOESM2_ESM.zip › Supplementary Data 2/training/Mcap--59-_jpg.rf.f50683c3dc853a168f65eea9d6e39e36.jpg]

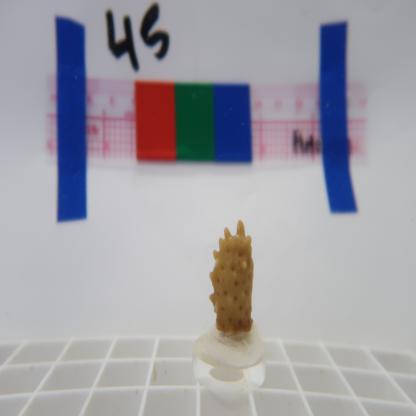

Supplement: Supplementary file 2 — Supplementary Information 2. [file 41598_2022_18653_MOESM2_ESM.zip › Supplementary Data 2/training/Mcap--13-_jpg.rf.3216d89bdd33a68a04d3cba7c3ec09f6.jpg]

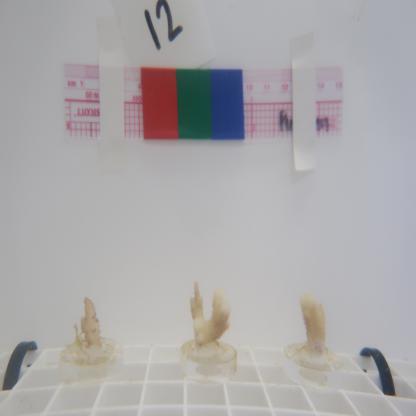

Supplement: Supplementary file 2 — Supplementary Information 2. [file 41598_2022_18653_MOESM2_ESM.zip › Supplementary Data 2/training/Pacuta--1-_jpg.rf.05cb41707e8827cfee615507c7ce3cbb.jpg]

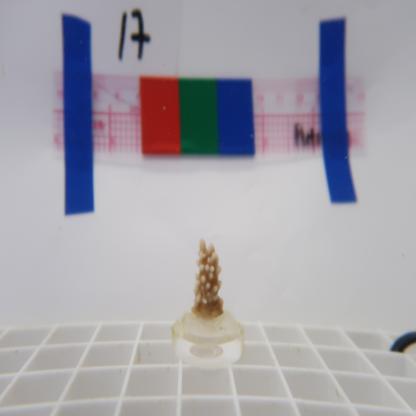

Supplement: Supplementary file 2 — Supplementary Information 2. [file 41598_2022_18653_MOESM2_ESM.zip › Supplementary Data 2/training/Mcap--17-_jpg.rf.54be5c997083ae767a6a7ae1c3d3b6b2.jpg]

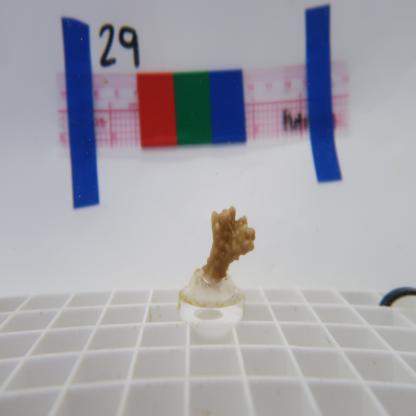

Supplement: Supplementary file 2 — Supplementary Information 2. [file 41598_2022_18653_MOESM2_ESM.zip › Supplementary Data 2/training/CIG_IMG013_jpg.rf.09c49adff3c1e1d050d6756e364df0bd.jpg]

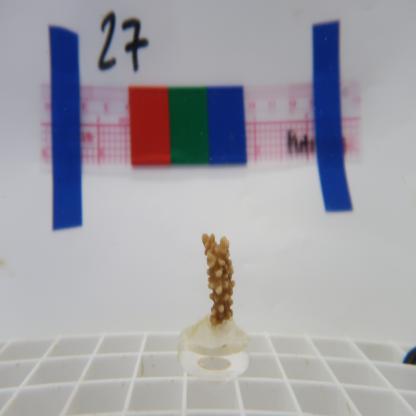

Supplement: Supplementary file 2 — Supplementary Information 2. [file 41598_2022_18653_MOESM2_ESM.zip › Supplementary Data 2/training/Mcap--16-_jpg.rf.fba06a68061a046225dd7907658e86aa.jpg]

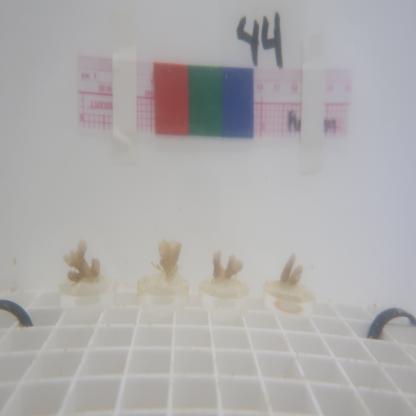

Supplement: Supplementary file 2 — Supplementary Information 2. [file 41598_2022_18653_MOESM2_ESM.zip › Supplementary Data 2/training/Pacuta--37-_jpg.rf.13591bae3a5a3075b37adcdaced9d6ab.jpg]

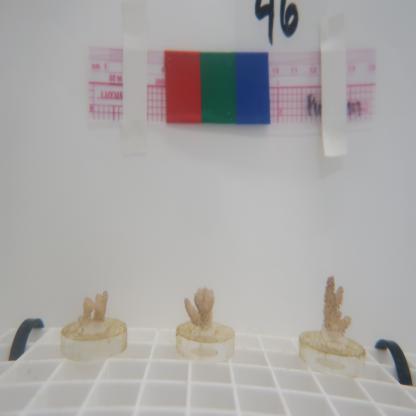

Supplement: Supplementary file 2 — Supplementary Information 2. [file 41598_2022_18653_MOESM2_ESM.zip › Supplementary Data 2/training/Pacuta--38-_jpg.rf.fd21e561f126353e52b45013011d4aef.jpg]

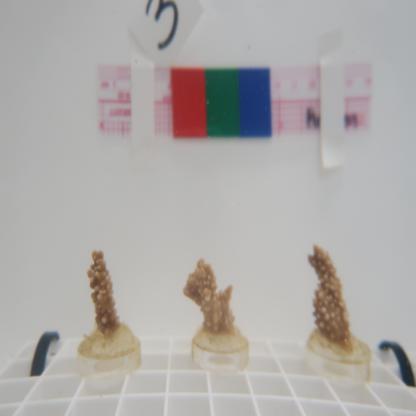

Supplement: Supplementary file 2 — Supplementary Information 2. [file 41598_2022_18653_MOESM2_ESM.zip › Supplementary Data 2/training/Mcap--3-_jpg.rf.b0ba9a87dd0081bdb55a7697c0ea7389.jpg]

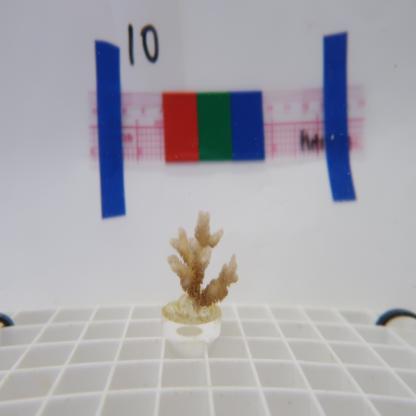

Supplement: Supplementary file 2 — Supplementary Information 2. [file 41598_2022_18653_MOESM2_ESM.zip › Supplementary Data 2/training/Pacuta--6-_jpg.rf.7f54282c685b654b0cdd2bed14bb1699.jpg]

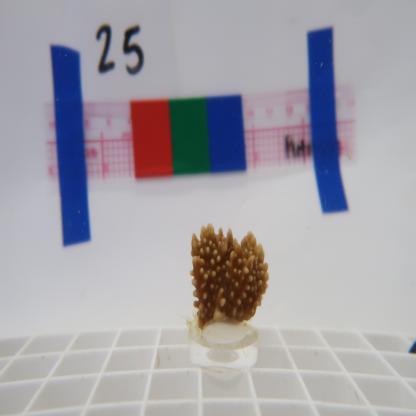

Supplement: Supplementary file 2 — Supplementary Information 2. [file 41598_2022_18653_MOESM2_ESM.zip › Supplementary Data 2/training/Mcap--15-_jpg.rf.0b58b9398d49ab7393ea02aea5287a41.jpg]

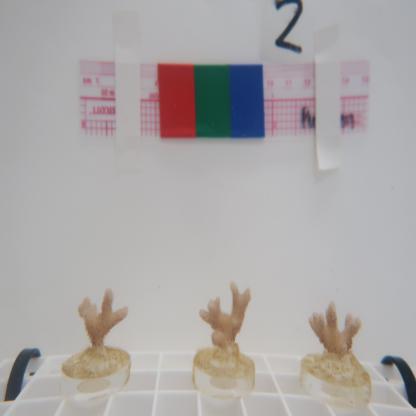

Supplement: Supplementary file 2 — Supplementary Information 2. [file 41598_2022_18653_MOESM2_ESM.zip › Supplementary Data 2/training/Pacuta--2-_jpg.rf.946d36d86d72fa960fd91fd784587025.jpg]

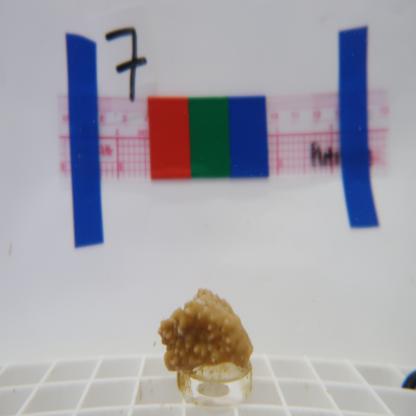

Supplement: Supplementary file 2 — Supplementary Information 2. [file 41598_2022_18653_MOESM2_ESM.zip › Supplementary Data 2/training/Mcap--5-_jpg.rf.eeba2280e45680ef7144fbd80756118b.jpg]

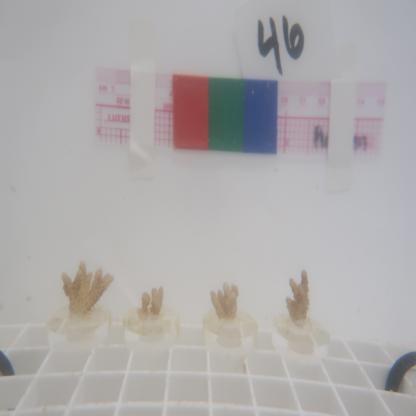

Supplement: Supplementary file 2 — Supplementary Information 2. [file 41598_2022_18653_MOESM2_ESM.zip › Supplementary Data 2/training/Pacuta--39-_jpg.rf.adb5466e94ffa63728ddafd0ac621a7c.jpg]

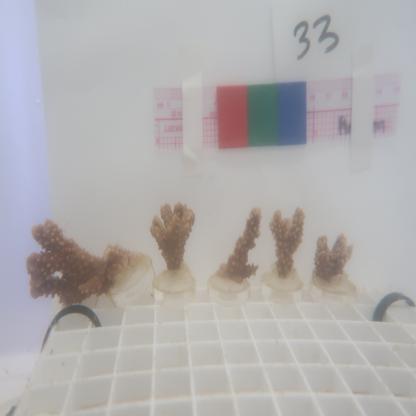

Supplement: Supplementary file 2 — Supplementary Information 2. [file 41598_2022_18653_MOESM2_ESM.zip › Supplementary Data 2/training/Mcap--58-_jpg.rf.b12bd973beb3de63ce8d1e81e308189f.jpg]

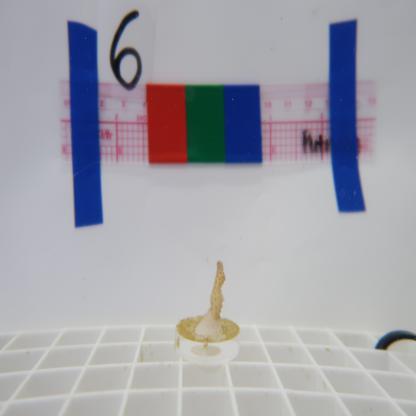

Supplement: Supplementary file 2 — Supplementary Information 2. [file 41598_2022_18653_MOESM2_ESM.zip › Supplementary Data 2/training/Pacuta--4-_jpg.rf.f3fa265a0720cecf458ebc2ae72a2b91.jpg]

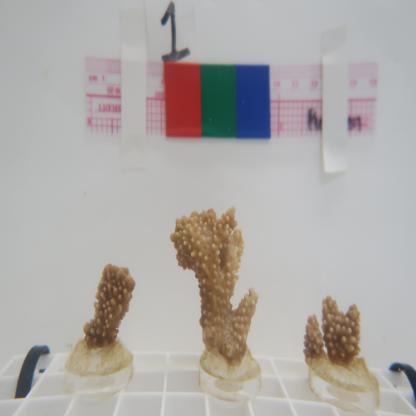

Supplement: Supplementary file 2 — Supplementary Information 2. [file 41598_2022_18653_MOESM2_ESM.zip › Supplementary Data 2/training/Mcap--2-_jpg.rf.90e4cd6f721f0dc17dae474548f986c8.jpg]

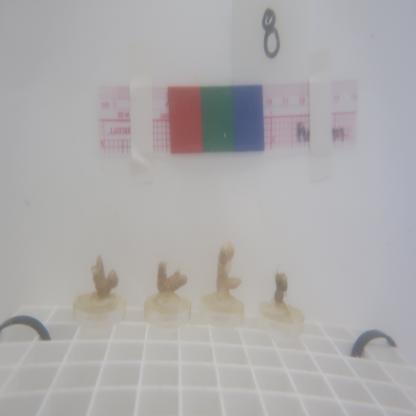

Supplement: Supplementary file 2 — Supplementary Information 2. [file 41598_2022_18653_MOESM2_ESM.zip › Supplementary Data 2/training/Pacuta--26-_jpg.rf.370640b29f6952b846c6783c681cf916.jpg]

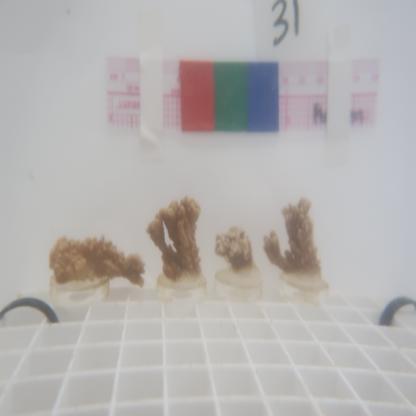

Supplement: Supplementary file 2 — Supplementary Information 2. [file 41598_2022_18653_MOESM2_ESM.zip › Supplementary Data 2/training/Mcap--33-_jpg.rf.8e56e66c78850a72f2467e48520493ae.jpg]

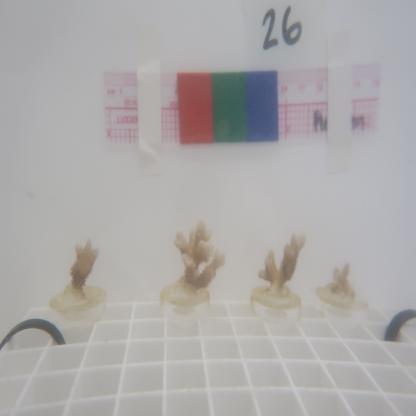

Supplement: Supplementary file 2 — Supplementary Information 2. [file 41598_2022_18653_MOESM2_ESM.zip › Supplementary Data 2/training/Pacuta--28-_jpg.rf.1d5d79f2f98629c8eedd50fa5158b5b6.jpg]

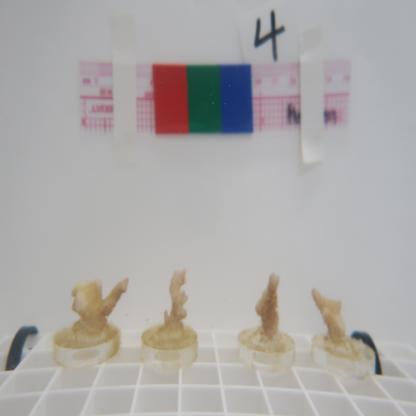

Supplement: Supplementary file 2 — Supplementary Information 2. [file 41598_2022_18653_MOESM2_ESM.zip › Supplementary Data 2/training/Pacuta--3-_jpg.rf.e298b8696d264a2db3e598778f769a9a.jpg]

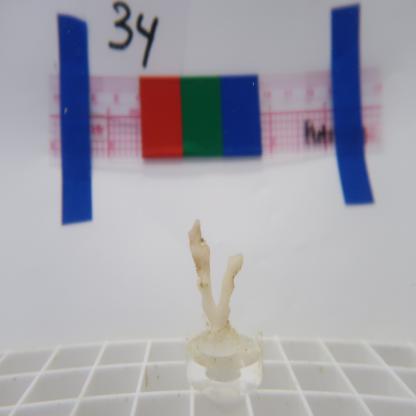

Supplement: Supplementary file 2 — Supplementary Information 2. [file 41598_2022_18653_MOESM2_ESM.zip › Supplementary Data 2/training/Pacuta--20-_jpg.rf.0bdc5f98657346e5c9dad868c3a75c5b.jpg]

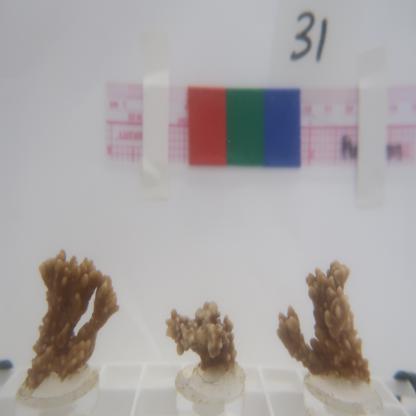

Supplement: Supplementary file 2 — Supplementary Information 2. [file 41598_2022_18653_MOESM2_ESM.zip › Supplementary Data 2/training/CIG_IMG015_jpg.rf.5b8cdb80d02e72e1728e71366da20d0f.jpg]

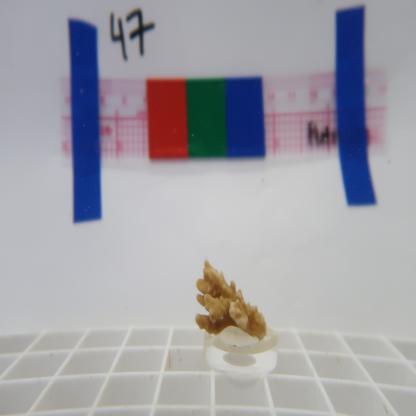

Supplement: Supplementary file 2 — Supplementary Information 2. [file 41598_2022_18653_MOESM2_ESM.zip › Supplementary Data 2/validation/Mcap--14-_jpg.rf.a669f0095ac55f95617db87ed32190ca.jpg]

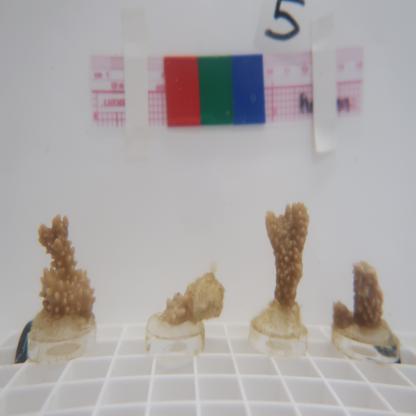

Supplement: Supplementary file 2 — Supplementary Information 2. [file 41598_2022_18653_MOESM2_ESM.zip › Supplementary Data 2/validation/Mcap--4-_jpg.rf.421184e6ce67dea800ca51710eed6da6.jpg]

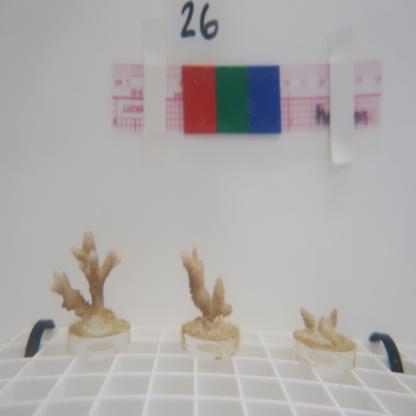

Supplement: Supplementary file 2 — Supplementary Information 2. [file 41598_2022_18653_MOESM2_ESM.zip › Supplementary Data 2/validation/Pacuta--27-_jpg.rf.0d26353ffbfd589e14b0fdebb266c13f.jpg]

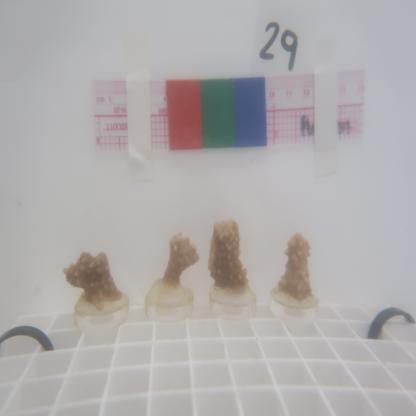

Supplement: Supplementary file 2 — Supplementary Information 2. [file 41598_2022_18653_MOESM2_ESM.zip › Supplementary Data 2/validation/Mcap--31-_jpg.rf.c82759a94755d47b8bafc3f190aa9511.jpg]

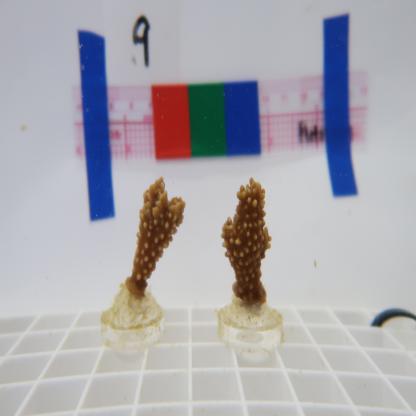

Supplement: Supplementary file 2 — Supplementary Information 2. [file 41598_2022_18653_MOESM2_ESM.zip › Supplementary Data 2/validation/Mcap--6-_jpg.rf.23e2386c7f090ed1002215aab0a7cf28.jpg]

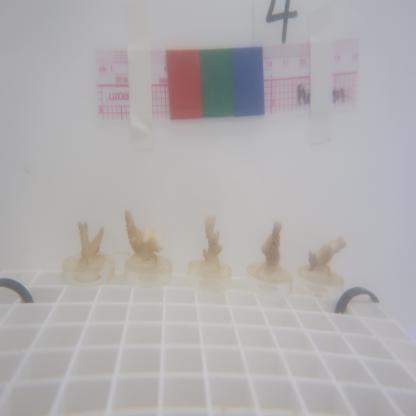

Supplement: Supplementary file 2 — Supplementary Information 2. [file 41598_2022_18653_MOESM2_ESM.zip › Supplementary Data 2/validation/Pacuta--19-_jpg.rf.7ee7ad145a7a79ef140d92ca3b034a9b.jpg]

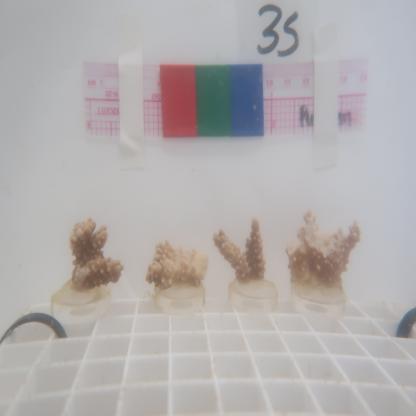

Supplement: Supplementary file 2 — Supplementary Information 2. [file 41598_2022_18653_MOESM2_ESM.zip › Supplementary Data 2/validation/Mcap--60-_jpg.rf.225ae3426334c505f9921db4bce45607.jpg]

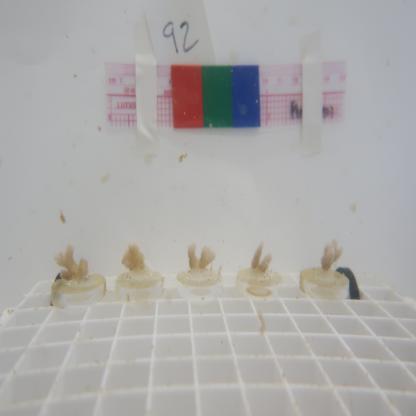

Supplement: Supplementary file 2 — Supplementary Information 2. [file 41598_2022_18653_MOESM2_ESM.zip › Supplementary Data 2/validation/CIG_IMG048_jpg.rf.203eeeb03fd57de3655394561eff3d86.jpg]

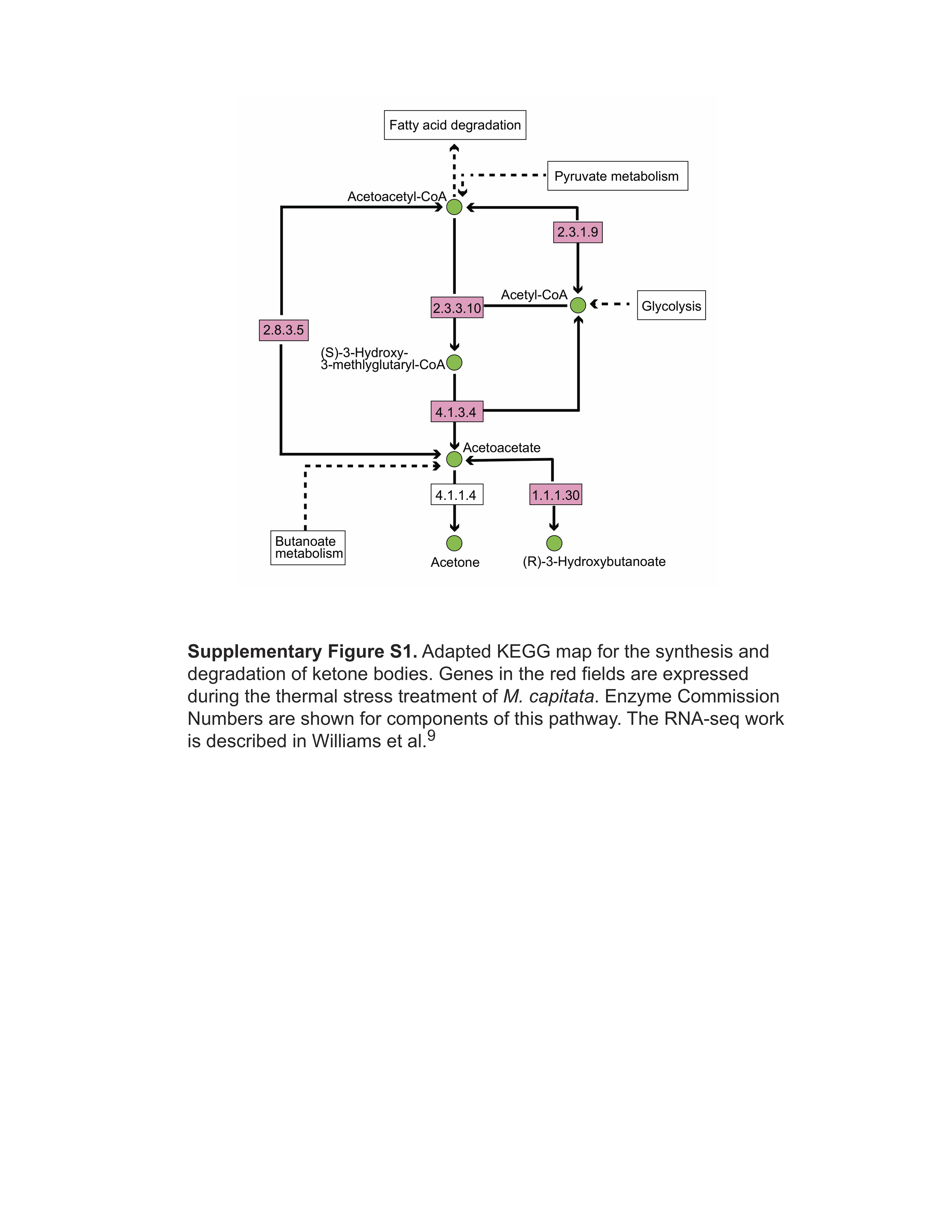

Supplement: Supplementary file 3 — Supplementary Information 3. [file 41598_2022_18653_MOESM3_ESM.jpg]
